# Osteocytes Serve as a Reservoir for Intracellular Persisting *Staphylococcus aureus* Due to the Lack of Defense Mechanisms

**DOI:** 10.3389/fmicb.2022.937466

**Published:** 2022-07-22

**Authors:** Marina Garcia-Moreno, Paul M. Jordan, Kerstin Günther, Therese Dau, Christian Fritzsch, Monika Vermes, Astrid Schoppa, Anita Ignatius, Britt Wildemann, Oliver Werz, Bettina Löffler, Lorena Tuchscherr

**Affiliations:** ^1^Institute of Medical Microbiology, Jena University Hospital, Jena, Germany; ^2^Department of Pharmaceutical/Medicinal Chemistry, Institute of Pharmacy, Friedrich Schiller University Jena, Jena, Germany; ^3^Leibniz Institute on Aging, Fritz Lipmann Institute, Jena, Germany; ^4^Institute of Orthopaedic Research and Biomechanics, University Medical Center Ulm, Ulm, Germany; ^5^Experimental Trauma Surgery, Department of Trauma, Hand and Reconstructive Surgery, Jena University Hospital, Friedrich Schiller University Jena, Jena, Germany

**Keywords:** *S. aureus*, osteocytes, osteoblasts, persistence, chronic osteomyelitis

## Abstract

Chronic staphylococcal osteomyelitis can persist for long time periods causing bone destruction. The ability of *Staphylococcus aureus* to develop chronic infections is linked to its capacity to invade and replicate within osteoblasts and osteocytes and to switch to a dormant phenotype called small colony variants. Recently, osteocytes were described as a main reservoir for this pathogen in bone tissue. However, the mechanisms involved in the persistence of *S. aureus* within these cells are still unknown. Here, we investigated the interaction between *S. aureus* and osteoblasts or osteocytes during infection. While osteoblasts are able to induce a strong antimicrobial response and eliminate intracellular *S. aureus*, osteocytes trigger signals to recruit immune cells and enhance inflammation but fail an efficient antimicrobial activity to clear the bacterial infection. Moreover, we found that extracellular signals from osteocytes enhance intracellular bacterial clearance by osteoblasts. Even though both cell types express Toll-like receptor (TLR) 2, the main TLR responsible for *S. aureus* detection, only osteoblasts were able to increase TLR2 expression after infection. Additionally, proteomic analysis indicates that reduced intracellular bacterial killing activity in osteocytes is related to low antimicrobial peptide expression. Nevertheless, high levels of lipid mediators and cytokines were secreted by osteocytes, suggesting that they can contribute to inflammation. Taken together, our results demonstrate that osteocytes contribute to severe inflammation observed in osteomyelitis and represent the main niche for *S. aureus* persistence due to their poor capacity for intracellular antimicrobial response.

## Introduction

*Staphylococcus aureus* osteomyelitis (OM) often develops to a chronic and destructive course that is extremely difficult to treat and can require long period of antibiotic therapies as well as surgical interventions (Fritz and McDonald, 2008; Kavanagh et al., 2018). Numerous antimicrobials cannot penetrate and reach adequate concentrations for bactericidal efficacy in bone compared to soft tissue (Thompson and Townsend, 2011; Tuchscherr et al., 2016; Alder et al., 2020). Furthermore, some bacteria internalize and survive within bone cells for a long period of time, which contributes to insufficient treatments (Tuchscherr et al., 2016; De Mesy Bentley et al., 2018; Yang et al., 2018; Muthukrishnan et al., 2019; Masters et al., 2021). Access to the intracellular environment protects this pathogen from the action of the host immune response and antimicrobials, promoting persistence (Tuchscherr et al., 2011; Horn et al., 2018). The intracellular adaptation of *S. aureus* in host cells is associated with an altered bacterial phenotype, the so-called small colony variants (SCVs; Proctor et al., 2006; Tuchscherr et al., 2010, 2020; Garcia et al., 2013; Proctor, 2019). SCVs form slow-growing and small colonies on agar plates and have reduced or absent pigmentation and virulence factors (Proctor et al., 2006). Additionally, SCVs exhibit altered drug resistance profiles and contribute to antibiotic treatment failure (Garcia et al., 2013; Kahl et al., 2016; Tuchscherr et al., 2016). Despite their lack of toxin expression, SCVs are capable of inducing strong host cell death, which associates this bacterial phenotype with significant morbidity and poor clinical outcome (Wickersham et al., 2017; Prince and Wong Fok Lung, 2020; Wong Fok Lung et al., 2020).

Although different studies have described that *S. aureus* can survive for long periods within osteoblasts (Kalinka et al., 2014; Ji et al., 2020; Bongiorno et al., 2021; Gunn et al., 2021; Marro et al., 2021), the mechanisms that may explain the persistence within bone cells are still unknown. In addition to osteoblasts, several studies have revealed osteocytes as possible niche for bacteria within bone tissue (De Mesy Bentley et al., 2018; Yang et al., 2018; Krauss et al., 2019; Masters et al., 2021). Recently, the persistence of *S. aureus* in bone tissue was associated with its capacity to invade the osteocyte lacuno-canalicular network (OLCN; Masters et al., 2021). However, the mechanisms that contribute to the development of *S. aureus* persistence and immune evasion in chronic osteomyelitis are still unknown. Moreover, the communication between osteoblasts and osteocytes during infection is poorly studied. Osteocytes communicate with osteoblasts and osteoclasts *via* diverse signaling molecules such as the RankL/OPG axis and the Sost/Dkk1/Wnt axis (Robling and Bonewald, 2020).

Host cells recognize the pathogen-associated molecular patterns (PAMPs) of *S. aureus via* different pattern-recognition receptors (PRRs), such as Toll-like receptors (TLRs) 1, 2, 6, and 9 and nucleotide-binding oligomerization domains (NODs)-1 and -2. PRR activation leads to the generation of cytokines (IL-1α, IL-1β, IFN-γ, TNF-α, IL-17A, IL-17F, and IL-22), chemokines (CXCL1, CXCL2, CXCL9, CXCL10, CXCL11, CCL27, and CCL20), and antibacterial responses, such as antimicrobial peptides and production of reactive oxygen species (ROS; Brandt et al., 2018b). Furthermore, the activation of TLR2 may cause production of lipid mediators (LMs; Jimenez et al., 2005). LMs are implicated in the inflammatory response and include prostaglandins (PGs) and leukotrienes (LTs), which are produced from arachidonic acid (AA, C20:4, ω-6) *via* cyclooxygenases (COX) and 5-lipoxygenase (LOX) pathways, respectively (Jordan et al., 2020).

To persist, *S. aureus* has developed diverse mechanisms to avoid recognition by, e.g., TLRs and the subsequent activation of antimicrobial host response (Joo and Otto, 2015; De Jong et al., 2019). These mechanisms have been well-described for several cells but for osteocytes little is known.

In this study, we compared the long-term survival of *S. aureus* in murine MC3T3-E1 osteoblasts and MLO-Y4 osteocytes. Our results reveal that osteocytes can induce a pro-inflammatory response to recruit immune cells to clear the infection but also contribute to bone destruction. Moreover, they lack efficient antimicrobial activity to kill intracellular bacteria. Consequently, osteocytes represent a possible reservoir for *S. aureus* long-term persistence during osteomyelitis.

## Materials and Methods

### Bacterial Strains

The *S. aureus* strains used in the present study are LS1 from a septic arthritis isolate (Ahmed et al., 2001) and SH1000 8325–4 with functional *rsbU* (Horsburgh et al., 2002). All bacterial colonies were grown on Columbia blood agar base plates (Oxoid™—Thermo Fisher Scientific, Waltham, United States).

### Preparation of Bacterial Suspension

Bacterial cultures were cultured in BHI (Oxoid™—Thermo Fisher Scientific, Waltham, United States) medium and incubated at 37°C at 160 rpm overnight. The next day, the OD was adjusted to an OD578 nm of 0.05. The bacterial suspension was incubated for 3 h at 37°C at 160 rpm to obtain bacteria in the logarithmic growth phase. Next, after two washing steps, the bacteria were adjusted to OD578 nm = 1. The number of bacteria or CFUs present at OD578 nm was determined as follows: 1 was determined by plating serial dilutions onto blood agar and counting after 24 h incubation at 37°C using ColonyQuant HD© (Schuett-biotech GmbH, Göttingen).

### Cell Culture

Mouse MC3T3-E1 osteoblast (CRL-2593, subclone 4, obtained from the American Type Culture Collection, Germany) and MLO-Y4 osteocyte (created by Dr. Bonewald, Kerafast Inc., Boston, United States) cell lines were used for all experiments. Osteoblast cells were cultured in αMEM medium (Biochrom, Berlin, Germany) supplemented with 10% FBS (Biochrom, Berlin, Germany) and 100 U/ml penicillin/streptomycin (Merck Millipore, Billerica, United States). Osteocyte αMEM medium was supplemented with 5% FBS and 5% iron-fortified FBS (Sigma-Aldrich, St. Louis, United States). Cells were seeded and grown until they were confluent. Medium was changed every 2–3 days. Inserts used for co-culture experiments were obtained from ThinCert™ (Greiner, Germany).

### Infections Assays

The monoculture and co-culture cell culture models followed the same infection protocol. Volumes were adapted depending on the type of experiment. When the confluence was 80%, the experiment was performed as follows: the grown cells were counted, and the amount of bacterial OD578 nm = 1 suspension needed for an MOI (multiplicity of infection) of 30 was calculated. Cells were washed, and invasion medium [αMEM supplemented with 5% HSA (CSL Behring, Pennsylvania, United States) and 2.5% HEPES (Biochrom GmbH, Berlin)] were added to each flask. Then, the cells were incubated for 90 min with *S. aureus* strains at 37°C and 5% CO_2_. After that, the cells were washed, and stop medium [αMEM supplemented with 10% FBS and 1% lysostaphin (WAK-chemie, Medical GmbH, Germany)] was added and incubated for 30 min at 37°C and 5% CO_2_ to kill all extracellular bacteria. Next, the cells were lysed by sterile ice-cold H_2_O. Afterward, the cells were scrapped and centrifuged at 5,000 rpm for 15 min at 4°C to release all intracellular bacteria. Serial dilutions were plated onto blood agar plates to count bacterial CFUs the next day. As an infection control, one flask of uninfected cells was treated in the same way as the infected cells. Of note, osteocytes represent 90–95% of the cells in bone. For our co-cultivation experiments, the relation 9:1 is not possible to keep *in vitro*. In all experiments, the number of osteocytes was three times higher than the number of osteoblasts. Furthermore, all results were adjusted to 1 × 10^6^ cells to compare both cells type.

### Persistence Assays

The monoculture and co-culture models followed the same infection protocol. Volumes were adapted depending on the type of experiment. When the confluence was 80%, the experiment was performed as follows: mouse MC3T3-E1 osteoblasts and MLO-Y4 osteocytes were infected with *S. aureus* strains, and the intracellular persistence capacity of the bacteria was traced for up to 7 days as we performed in previous studies (Tuchscherr et al., 2011, 2015). In this model, we can differentiate the acute from the chronic phase of the infection. During the acute phase, we observed high release of cytokines and homogeneous bacterial phenotype while the chronic phase was characterized by SCVs appearance and decrease in immune response (Tuchscherr et al., 2011).

To track the intracellular persistence of *S. aureus* in bone cells, cells were counted and lysed at different time points to determine the number of intracellular bacteria. Cells were washed two times and lysed after 90 min of infection (T0), 2 days after infection (T2), 4 days after infection (T4), and 7 days after infection (T7). Cell lysis was performed at each time point after the stopping medium treatment. Cultured medium was added after the stopping medium treatment, which was performed every 2 days, in each non-lysed cell culture flask. Co-culture experiments were performed as described for the Infection assay.

### Counting SCVs

Normal and SCV colony phenotypes were counted on blood agar plates. SCV formation was monitored by incubating the blood agar plates for 24, 48, and 72 h for each time point.

### Conditioned Medium Experiments

For these experiments, conditioned medium from infected or uninfected control cells was used (Supplementary Figure S5). Briefly, in the first step, cells were cultured with or without *S. aureus* for 24 h to obtain conditioned medium. The medium of the cells was removed and filtered (0.2 μm filter SARSTEDT AG & Co, Germany). This medium was called the “conditioned” medium. The sterility of the conditioned medium was controlled by streaking 100 μl of the medium onto a blood agar plate and incubating for 24 h at 37°C. The conditioned medium obtained from non-infected cells was called control CM whereas the conditioned medium obtained from infected cells was called infected CM. The conditioned medium was then added to fresh cell cultures. Cells with conditioned medium were cultured for 24 h at 37°C and 5% CO2. After 24 h, the cells were washed with PBS 1X and infected with *S. aureus* LS1 as described above. After 90 min of incubation, the cells were washed and treated with lysostaphin to kill extracellular bacteria for 30 min. Thirty minutes later, the cells were washed and lysed to recover intracellular bacteria.

### TLR2 Measurement by Flow Cytometry Analysis

MC3T3-E1 osteoblasts and MLO-Y4 osteocytes were infected with *S. aureus* LS1 strain MOI 30 for 90 min. After that, extracellular bacteria were killed for 30 min, and full medium was added. Infected cells were incubated for 24 h. Next, cells were detached by treatment with trypsin for 5 min at 37°C. Afterward, the cells were centrifuged at 400 *g* and 4°C for 10 min. To determine viability, the cells were resuspended in 30 μl (1:500) Zombie Aqua Fixable Viability Kit (BioLegend, San Diego, CA, United States) for 5 min. Non-specific antibody binding was blocked by rat serum (5 min, 4°C) prior to antibody staining. Then, the cells were stained with fluorochrome-labelled antibody mixtures for 20 min at 4°C. APC recombinant mouse anti-mouse/human CD282 (TLR2) was used to detect TLR2 (0.6 μg/test, clone QA16A01, Biolegend). Fluorescent staining for flow cytometric analysis of cells was performed in FACS buffer (PBS plus 0.5% BSA, 5 mM EDTA, and 0.1% sodium azide). After the staining, cells were fixed with 4% paraformaldehyde (15 min, 4°C). Cells were analyzed using BD LSR Fortessa (BD Biosciences), and data were analyzed using FlowJo X Software (BD Biosciences).

### Agr Functionality (CAMP Assay)

Agr functionality was detected from *S. aureus* LS1 and SH1000 by plating the strains perpendicularly to RN4220, which produces only β-hemolysin. This toxin induces on other strains the synthesis of δ-hemolysin, but inhibits lysis of erythrocytes by α-hemolysin. RN4220 strain was spread, directly from the overnight culture, in the middle of the blood agar plate before the strains of interest. The plate was incubated for 5 h at 37°C. After that, *S. aureus* LS1 and SH1000 overnight cultures were plated with a swab perpendicularly to RN4220. *Staphylococcus aureus* Mw2 and USAΔ*agr* strains were used as positive and negative controls, respectively (Traber et al., 2008).

### Expression of *psmα*

For RNA isolation of *in vitro* shaking cultures, *S. aureus* was grown in BHI medium after inoculation to OD578nm = 0.05 from overnight cultures. After 5 h, 1 ml of the bacterial culture was mixed with 1 ml of RNAprotect Bacteria Reagent (Qiagen), for 5 min at RT and centrifuged for 10 min at 10,000 rpm. The pellet was resuspended in 1 ml of RNApro Solution (MP) and transferred into BashingBead Lysis Tubes (Zymo Research). Homogenization was performed in a FastPrep (MP) at a setting of 6.5 for 45 s. The supernatants were mixed with 70% EtOH and RNA was isolated by using a Total RNA Kit [peqGOLD (Peqlab)], including the digestion of genomic DNA with DNase (Ambion/ThermoFisher).RNA was transcribed into complementary DNA (cDNA) by using a QuantiNova Reverse Transcription Kit (Qiagen), following manufacturer’s instructions. The Real-time PCR (RT-PCR) was performed using a Rotor-Gene SYBR Green PCR Kit (Qiagen). The reaction mixture was incubated at 95°C for 5 min, followed by 40 cycles at 95°C for 5 s and at 60°C for 10 s using a Rotor-Gene Q (Qiagen). Expression rates, efficiencies, and melting curves were analyzed with Rotor-Gene Q Series software (Qiagen). The different primers used to analyze the expression of bacterial genes are listed in Supplementary Table S1. Fold changes in expression were calculated as described elsewhere (Pfaffl, 2001). The *gyrB* gene was used as a reference.

### Proteomics Assay

Cells were infected as described above, and 24 h post infection, the supernatants were filter sterilized and stored in low protein binding tubes (Protein LoBind® Tubes, Eppendorf).

### Sample Preparation for Proteomics Analysis

For proteomics analysis, lysis buffer (final concentration of 5% SDS, 100 mM HEPES and 50 mM DTT) was added to 12.5 μl supernatant. The samples were sonicated (Bioruptor Plus, Diagenode, Belgium) for 10 cycles (30 s ON/60 s OFF) at a high setting at 20°C, followed by boiling at 95°C for 5 min. Reduction was followed by alkylation with iodoacetamide (final concentration 15 mM) for 30 min at room temperature in the dark. Samples were acidified with phosphoric acid (final concentration 2.5%), and 165 μl S-trap binding buffer was added (100 mM TEAB, 90% methanol). Samples were bound on S-trap micro spin columns (Protifi) and washed three times with binding buffer. Trypsin in 50 mM TEAB pH 7.55 was added to the samples (1 μg per sample) and incubated for 1 h at 47°C. The samples were eluted in three steps with 50 mM TEAB pH 7.55, elution buffer 1 (0.2% formic acid in water) and elution buffer 2 (50% acetonitrile and 0.2% formic acid). The eluates were dried using a speed vacuum centrifuge (Eppendorf Concentrator Plus, Eppendorf AG, Germany). The samples were resuspended in Evosep buffer A (0.1% formic acid in water) and sonicated (Bioruptor Plus, Diagenode, Belgium) for 3 cycles (60 s ON/30 s OFF) at a high setting at 20°C.

The samples were loaded on Evotips (Evosep) according to the manufacturer’s instructions. In short, Evotips were first washed with Evosep buffer B (acetonitrile, 0.1% formic acid), conditioned with 100% isopropanol and equilibrated with Evosep buffer A. Afterward, the samples were loaded on the Evotips and washed with Evosep buffer A. The loaded Evotips were topped up with buffer A and stored until the measurement.

### LC–MS Data Independent Analysis

Peptides were separated using the Evosep One system (Evosep, Odense, Denmark) equipped with a 15 cm × 150 μm i.d. packed with a 1.9 μm Reprosil-Pur C18 bead column (Evosep Endurance, EV-1106, PepSep, Marslev, Denmark). The samples were run with a pre-programmed proprietary Evosep gradient of 44 min (30 samples per day) using water and 0.1% formic acid and solvent B acetonitrile and 0.1% formic acid as solvents. The LC was coupled to an Orbitrap Exploris 480 (Thermo Fisher Scientific, Bremen, Germany) using PepSep Sprayers and a Proxeon nanospray source. The peptides were introduced into the mass spectrometer *via* a PepSep Emitter 360-μm outer diameter × 20-μm inner diameter, heated at 300°C, and a spray voltage of 2.2 kV was applied. The injection capillary temperature was set at 300°C. The radio frequency ion funnel was set to 30%. For data independent analysis (DIA) data acquisition, full scan mass spectrometry (MS) spectra with a mass range of 350–1,650 m/z were acquired in profile mode in the Orbitrap with a resolution of 120,000 FWHM. The default charge state was set to 2+, and the filling time was set at a maximum of 60 ms with a limitation of 3 × 10^6^ ions. DIA scans were acquired with 40 mass window segments of differing widths across the MS1 mass range. Higher collisional dissociation fragmentation (stepped normalized collision energy; 25, 27.5, and 30%) was applied, and MS/MS spectra were acquired with a resolution of 30,000 FWHM with a fixed first mass of 200 m/z after accumulation of 1 × 10^6^ ions or after filling time of 45 ms (whichever occurred first). Data were acquired in profile mode. For data acquisition and processing of the raw data, Xcalibur 4.4 (Thermo) and Tune version 3.1 were used.

### Proteomic Data Processing

Data independent analysis raw data were analyzed using the directDIA pipeline in Spectronaut (v.13, Biognosysis, Switzerland). The data were searched against a species-specific (*Homo sapiens*, 20.186 entries) and contaminant (247 entries) SwissProt database. The data were searched with the following variable modifications: oxidation (M) and acetyl (protein N-term). A maximum of two missed cleavages for trypsin and five variable modifications were allowed. The identifications were filtered to satisfy an FDR of 1% at the peptide and protein levels. Relative quantification was performed in Spectronaut for each paired comparison using the replicate samples from each condition. The data (candidate table) and data reports (protein quantities) were then exported, and further data analyses and visualization were performed with R studio using in-house pipelines and scripts. To select significant proteins, a log2FC cut-off of 0.58 and a *q* value < 0.05 were defined. The proteomics data have been uploaded to the ProteomeXchange *via* the PRIDE database.^1^

### Cell Dead Assay by Flow Cytometry Analysis

Cells were infected as described above but with different MOIs: 10, 30, 50, 70, and 100. Twenty-four hours post infection, the supernatant and unattached cells were collected in the same tube. The tubes were centrifuged at 180 × *g* for 5 min. The supernatant was discarded, and the cellular pellet was resuspended in 500 μl of PBS. Cell death was measured by flow cytometry by staining the cells with 50 μl of propidium iodine.

### Measurement of IL-6 and LL-37 (Cathelicidin Antimicrobial Peptide, CAMP) by ELISA

Cells were infected as described above, and 24 h post-infection, the supernatants were collected and centrifuged at 1,000 rpm for 5 min to exclude cellular derives. ELISA for IL-6 (DuoSet ELISA, 5 Plates from R&D Systems, Germany) and cathelicidin antimicrobial peptide (CAMP; assayGenie mouse CAMP ELISA kit) was performed following the manufacturer’s instructions. IL-6 and CAMP secretion was measured by a microplate reader at 450 and 570 nm (TECAN Infinite® 200 PRO).

### Lipid Meditators Measured by UPLC-MS–MS

Cells were infected as described above. After a 24 h incubation period, the supernatants (3 ml) were transferred to 6 ml of ice-cold methanol containing deuterium-labelled internal standards (200 nM d8-5S-HETE, d4-LTB_4_, d5-LXA_4_, d5-RvD2, d4-PGE_2_, and 10 mM d8-AA; Cayman Chemical/Biomol GmbH, Hamburg, Germany) to facilitate quantification and sample recovery. Sample preparation was conducted by adapting published criteria (Werz et al., 2018). In brief, samples were kept at −20°C overnight to allow protein precipitation. After centrifugation (1,200 *g*, 4°C, 10 min), 27 ml acidified H_2_O was added (final pH = 3.5), and samples were subjected to solid phase extraction. Solid phase cartridges (Sep-Pak ® Vac 6 cc 500 mg/6 ml C18; Waters, Milford, MA, United States) were equilibrated with 6 ml methanol and 2 ml H_2_O before samples were loaded onto columns. After washing with 6 ml H_2_O and an additional 6 ml *n*-hexane, LMs were eluted with 6 ml methyl formate. Finally, the samples were dried using an evaporation system (TurboVap LV, Biotage, Uppsala, Sweden) and resuspended in 150 μl methanol/water (50/50, v/v) for UPLC/MS–MS automated injections. LM profiling was analyzed with an Acquity UPLC system (Waters, Milford, MA, United States) and a QTRAP 5500 Mass Spectrometer (Sciex, Darmstadt, Germany) equipped with a Turbo V Source and electrospray ionization. LM was eluted using an ACQUITY UPLC BEH C18 column (1.7 μm, 2.1 × 100 mm; Waters, Eschborn, Germany) at 50°C with a flow rate of 0.3 ml/min and a mobile phase consisting of methanol/water/acetic acid of 42:58:0.01 (v/v/v) that was ramped to 86:14:0.01 (v/v/v) over 12.5 min and then to 98:2:0.01 (v/v/v) for 3 min (Werner, 2019). The QTrap 5500 was operated in negative ionization mode using scheduled multiple reaction monitoring (MRM) coupled with information-dependent acquisition. The scheduled MRM window was 60 s, optimized LM parameters were adopted (Colas et al., 2014) and the curtain gas pressure was set to 35 psi. The retention time and at least six diagnostic ions for each LM were confirmed by means of an external standard (Cayman Chemical/Biomol GmbH, Hamburg, Germany). Quantification was achieved by calibration curves for each LM. Linear calibration curves were obtained for each LM and gave *r*^2^ values of 0.998 or higher (for fatty acids 0.95 or higher). Additionally, the limit of detection for each targeted LM was determined (Werner, 2019).

### Inhibition of TLR2

MC3T3-E1 osteoblasts and MLO-Y4 osteocytes were pre-treated with the TLR 2 inhibitor MMG-11 (50 μM, solved in DMSO, Biozol, Eching, Germany) for 1 h. Afterward, cells were infected with *S. aureus* LS1 strain MOI 30 for 90 min. Extracellular bacteria were killed for 30 min with lysostaphin as explained in infection assays, and full medium was added. Infected cells were incubated for 24 h. Next, supernatants were collected and LL-37 was measured by ELISA (see measurement of LL-37 by ELISA in Material and Methods). Cells were treated with sterile ice-cold H_2_O. Afterward, the cells were scrapped and centrifuged at 5,000 rpm for 15 min at 4°C to release all intracellular bacteria. Serial dilutions were plated onto blood agar plates to count bacterial CFUs on the next day. Control cells were pre-treated with DMSO only.

### Statistical Analysis

Data analysis of all results was performed by using GraphPad Prism 6.0 (GraphPad, La Jolla, CA, United States). All experiments were repeated at least three times. Different statistical analyses were performed according to the type of experiment described in each figure caption in the results. To compare several groups, one-way ANOVA was performed. An unpaired *t*-test was performed to analyze the difference between two groups. The statistical analysis of two variables, such as different time points and strains, was performed by using a two-way ANOVA test.

## Results

### *Staphylococcus aureus* Predominantly Persists in Osteocytes Than in Osteoblasts

**Figure 1:**
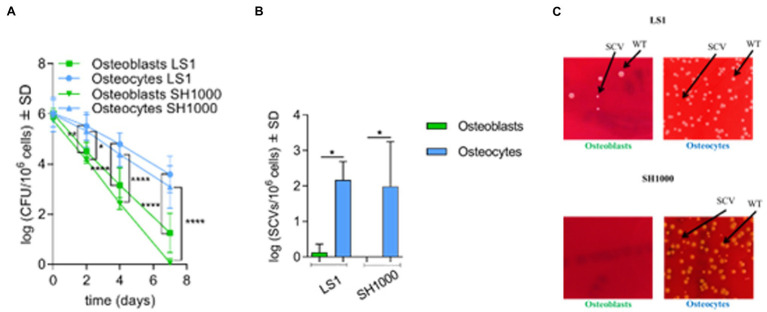
*Staphylococcus aureus* predominantly persists in osteocytes than osteoblasts. **(A)** Intracellular bacteria (log CFU/10^6^ cells) recovered from infected osteoblasts and osteocytes at different time points during long-term persistence. Osteoblasts LS1; *n* = 6, osteocytes LS1; *n* = 7, osteoblasts SH1000; *n* = 6 and osteocytes SH1000; *n* = 3; **(B)** SCV formation (log SCVs/10^6^ cells) on day 7 for *S. aureus* LS1 and SH1000 strains in osteoblasts and osteocytes. **(C)** SCV formation on day 7 on blood agar plates. *S. aureus* LS1 strain within; (i) osteoblasts and (ii) osteocytes. *Staphylococcus aureus* SH1000 strain within; (iii) osteoblasts; and (iv) osteocytes; *n* = 3. Differences were analyzed by using two-way and one-way ANOVA, respectively, with Tukey’s multiple comparison test; *p < 0.05, **p < 0.01, and ****p < 0.0001. The bars and whiskers represent the means ±SD of independent experiments.

To investigate *S. aureus* long-term persistence, MC3T3-E1 osteoblasts and MLO-Y4 osteocytes were infected with two independent *S. aureus* strains (LS1 or SH1000) for 90 min. Intracellular bacteria were counted after 90 min (day 0) and on days 2, 4, and 7 post infection. Both strains were included to investigate whether the interaction between bone cells and *S. aureus* is influenced by the genetic background and/or virulence profile of the bacteria. *Staphylococcus aureus* SH1000 expressed higher Agr, a quorum sensing system responsible of the regulation of virulence factors, and phenol soluble modulins (PSMs, a virulence factor regulated by Agr) than *S. aureus* LS1 (Supplementary Figure S1; Siegmund et al., 2021). The viability of both types of bone cells was not affected during the experimental period (Supplementary Figure S2 A). Moreover, no significant differences in bacterial internalization were observed between osteoblasts and osteocytes, suggesting that *S. aureus* invades both cell lines in a similar manner (Supplementary Figure S2 B). However, significant differences in the recovered intracellular bacteria were found after days 2, 4, and 7 when osteoblasts and osteocytes were compared (Figure 1A). *Staphylococcus aureus* LS1, as well as SH1000, were able to persist within osteocytes in significantly higher numbers than within osteoblasts. Of note, *S. aureus* SH1000 was fully cleared from osteoblasts after 7 days compared to *S. aureus* LS1. Based on our results, the virulence bacterial profile may influence the interaction between *S. aureus* and bone cells (Supplementary Figure S1).

The presence of SCVs is associated with the persistence and long-term survival of *S. aureus* (Tuchscherr et al., 2020). The appearance of SCVs was investigated at each time point during the long-term cell culture model for both types of bone cells. SCV formation increased along with elevated persistence in particular in osteocytes. While at days 0 and 2, all the colonies counted had normal colony phenotypes, on day 4, large wild-type and small SCV colonies were present on the blood agar plates. From day 4 to day 7, SCV formation increased significantly (Supplementary Figure S3; Figures 1B,C). Of interest, a high number of SCVs were found in osteocytes for both *S. aureus* strains at day 7 (Figures 1B,C; Supplementary Figure S3), indicating that SCV formation was promoted mainly in osteocytes in a strain-independent manner as previously described (Yang et al., 2018).

### Elevated Persistence of *Staphylococcus aureus* Occurs in Osteocytes During Co-cultivation With Osteoblasts

**Figure 2:**
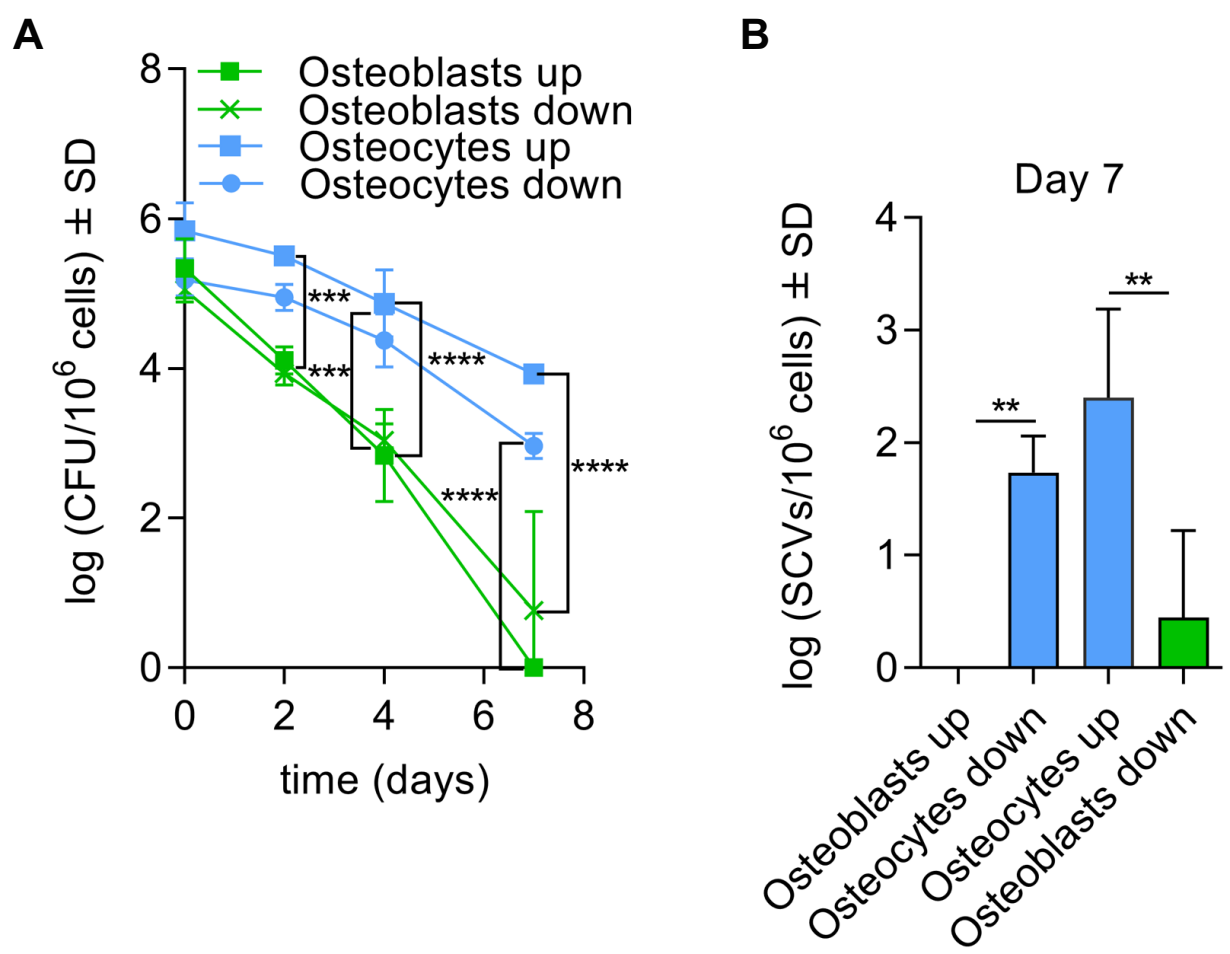
Elevated persistence of *Staphylococcusaureus* occurs in osteocytes during co-cultivation with osteoblasts. **(A)** Intracellular bacteria (log CFU/106 cells) recovered from infected osteoblasts and osteocytes with *S. aureus* LS1 strain at different time points and both possible orientations in transwell experiments (co-cultivation); **(B)** SCV (log SCVs/106 cells) formation of *S. aureus* LS1 strain for both possible orientations. Statistical analysis was performed using two-way ANOVA with Tukey’s multiple comparison test; **p < 0.01, ***p < 0.001, and ****p < 0.0001. The bars and whiskers represent the means ±SD of different independent experiments; *n* = 3.

To investigate the main cell type in bone tissue for long-term persistence a transwell system was established to infect co-cultivated osteoblasts and osteocytes cells with *S. aureus* LS1 or SH1000 strains (Supplementary Figure S4). The size of the transwell pore (3 μm) was selected to allow the transmigration of *S. aureus* (up to 1 μm) between both types of cells. To exclude a gravity effect, the experiments were performed in two possible orientations (Supplementary Figure S4). Both experimental setups showed similar results. *S. aureus* persisted (Figure 2A) and formed more SCVs after 7 days post infection (Figure 2B) in osteocytes than in osteoblasts independent of the orientation and *S. aureus* strain used (Supplementary Figures S5 A,B).

### Osteocytes Trigger Osteoblasts to Eliminate Intracellular Bacteria but Not Vice Versa

To investigate whether possible crosstalk between osteocytes and osteoblasts may interfere with the ability of *S. aureus* to survive intracellularly, experiments with conditioned medium were performed (Supplementary Figure S6). Conditioned medium contains numerous secreted enzymes, growth factors, cytokines and hormones or other soluble mediators that regulate cell–cell interactions. Thus, the conditioned medium from infected bone cells was used to incubate fresh cells prior to a new infection.

**Figure 3:**
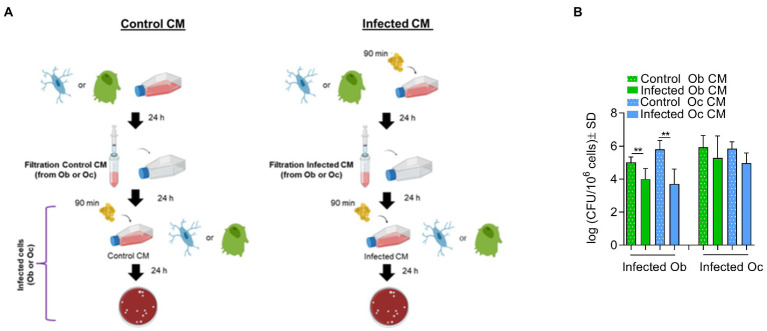
Osteocytes trigger osteoblasts to eliminate intracellular bacteria but not vice versa. **(A)** Model of infection. **(B)** Infected osteoblast (Ob) or osteocytes (Oc) cultured with control CM or infected CM were lysed and the numbers of intracellular bacteria were quantified by serial dilutions on blood agar plates (log CFU/106 cells). Statistical analysis was performed using the unpaired t-test for each pair; **p < 0.01. The bars and whiskers represent the means ±SD of different independent experiments; *n* = 6, *n* = 4, *n* = 4, and *n* = 5, respectively. CM, conditioned medium.

Osteoblasts or osteocytes were incubated for 24 h with conditioned medium (CM) taken from either uninfected cells (control CM) or cells previously infected with *S. aureus* LS1 (infected CM). After 24 h, CFU quantification revealed significant differences when osteoblasts were incubated prior to infection with infected CM from osteoblasts or osteocytes (Figure 3) compared to cells cultured with control CM. In particular, when osteoblasts were cultured with infected CM, the number of intracellular bacteria was significantly reduced. In contrast, no significant differences were observed between osteocytes cultured with control and infected CM (Figure 3). Similar results were obtained with the *S. aureus* SH1000 strain (Supplementary Figure S6). The incubation of the cells with the CM had no effect on cell viability (Supplementary Table S2). Therefore, the reduced CFU count was not due to reduced osteoblast viability. However, we could not exclude a possible effect of CM on cellular functionality of osteoblasts that may affect the persistence of *S. aureus* within osteoblasts. Next, we investigated whether the reduced number of intracellular CFUs within osteoblasts cultured with infected CM was due to an increased intracellular elimination (Table 1).

**Table 1:** Quantification of intracellular bacteria from conditioned cells. Intracellular clearance was calculated for each experiment (I_B_-T_B_).

		Initial added bacteria I_B_	Total recovered bacteria T_B_ = Extra_B_ + Intra_B_	Eliminated bacteria I_B_-T_B_	Comparison between control and conditioned cells Unpaired *t*-test (*p* value)
Osteoblasts	Control osteoblasts CM	7.83	7.57	0.26	^**^ *p* < 0.01
	Infected osteoblasts CM	7.76	6.21	1.55	
	Control osteocytes CM	7.65	7.65	−0.00	^***^ *p* < 0.001
	Infected osteocytes CM	7.6	4.71	2.89	
Osteocytes	Control osteocytes CM	8.05	8.78	−0.73	NS
	Infected osteocytes CM	8.02	7.91	0.11	
	Control osteoblasts CM	7.96	8.82	−0.86	NS
	Infected osteoblasts CM	7.91	7.99	−0.08	

I_B_, mean of the initial amount added of *Staphylococcus aureus* LS1 (expressed in log CFU/ml). T_B_, mean of the total amount of extracellular and intracellular *Staphylococcus aureus* LS1 strain recovered (T_B_ = Extra_B_ + Intra_B_; expressed in log CFU/ml). Statistical analysis was performed using the unpaired *t*-test for each pair; *n* = 4–6. **p < 0.01 and ***p < 0.001.

To quantify the intracellular killing, the elimination factor was calculated for each condition by comparing the initial added bacteria to the total recovered bacteria (Table 1). A value of “0” or around “0” indicates nor or poor elimination, and a value higher than “1” suggests intracellular killing. According to these calculations, we found that the bacteria were more efficiently killed in osteoblasts cultured with CM from infected osteoblasts (**p < 0.01) and osteocytes (***p < 0.01) than in CM from control osteoblasts (Table 1). However, no differences were observed for osteocytes treated with control or conditioned CM.

### Osteocytes Express Lower Levels of TLR2 Compared to Osteoblasts

Next, we examined possible underlying mechanisms of enhanced elimination of intracellular *S. aureus* in osteoblasts. *S. aureus* is recognized by osteoblasts *via* TLR2 (Josse et al., 2015), which triggers the activation of MyD88-dependent transcription factors such as NF-kB, AP1, and CREB to generate cytokines, chemokines, and antimicrobial effectors (Brandt et al., 2018a).

**Figure 4:**
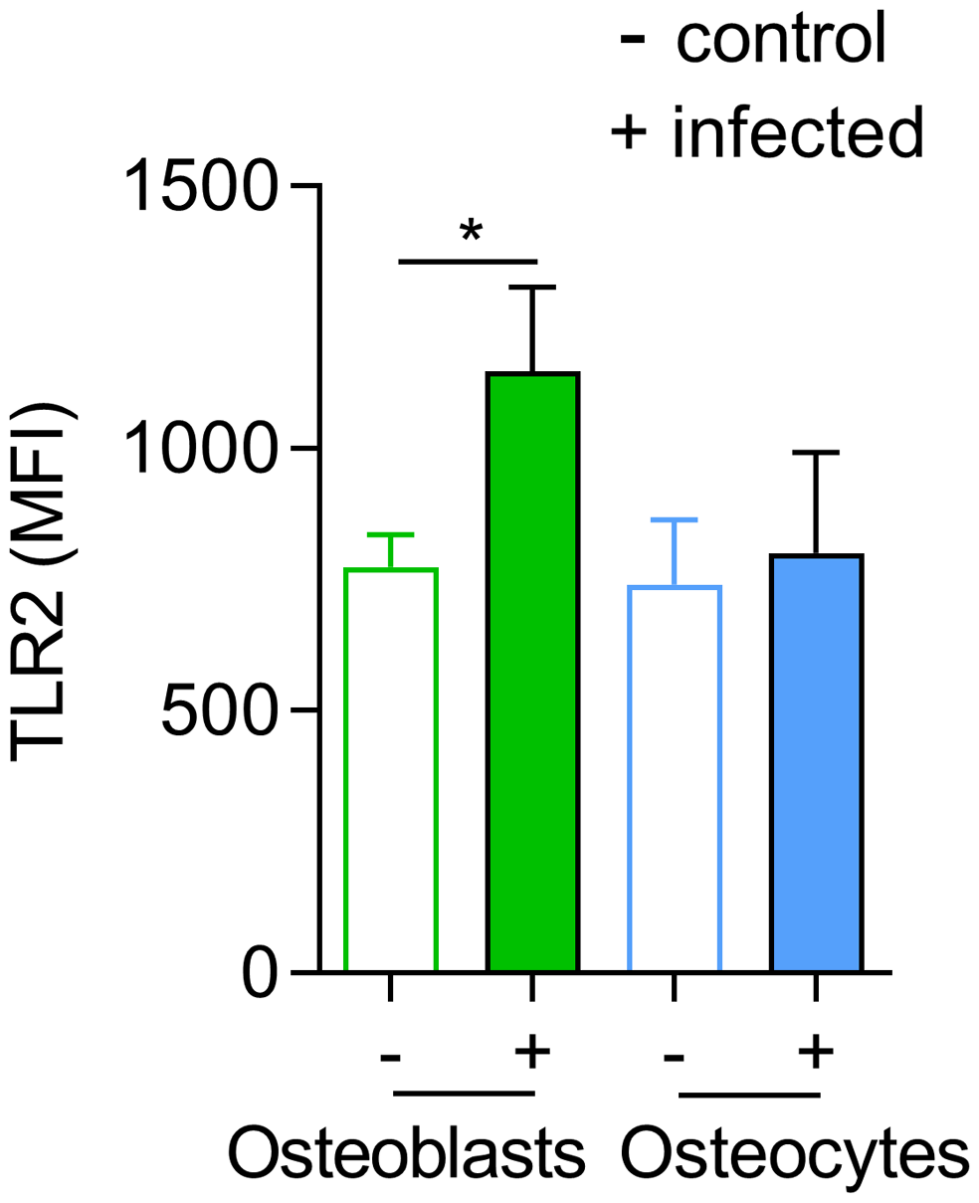
TLR2 expression is not altered in infected osteocytes. Influence of *Staphylococcus aureus* on osteoblasts and osteocytes surface TLR2 expression. Expression of TLR2 among living cells was analyzed by flow cytometry. The mean fluorescence intensity (MFI) of TLR2 was determined. Statistics are calculated with raw data (MFI), infected vs. uninfected and a statistical analysis was performed using the unpaired *t*-test for each pair unpaired *t*-test; *p < 0.05. The bars and whiskers represent the means ±SD of different independent experiments; *n* = 3. −: control cells and +: infected cells.

To investigate whether the differences between bacterial elimination by osteoblasts and osteocytes were due to differences in TLR2 receptors, the expression of this receptor was investigated by flow cytometry in infected and non-infected cells as described in material and methods (Figure 4). A significant increase in the levels of TLR2 expression was observed in osteoblasts upon infection but no differences were observed in osteocytes (Figure 4).

### Osteocytes Fail to Eliminate Intracellular Bacteria Due to Poor Production of Antimicrobial Peptides

**Figure 5:**
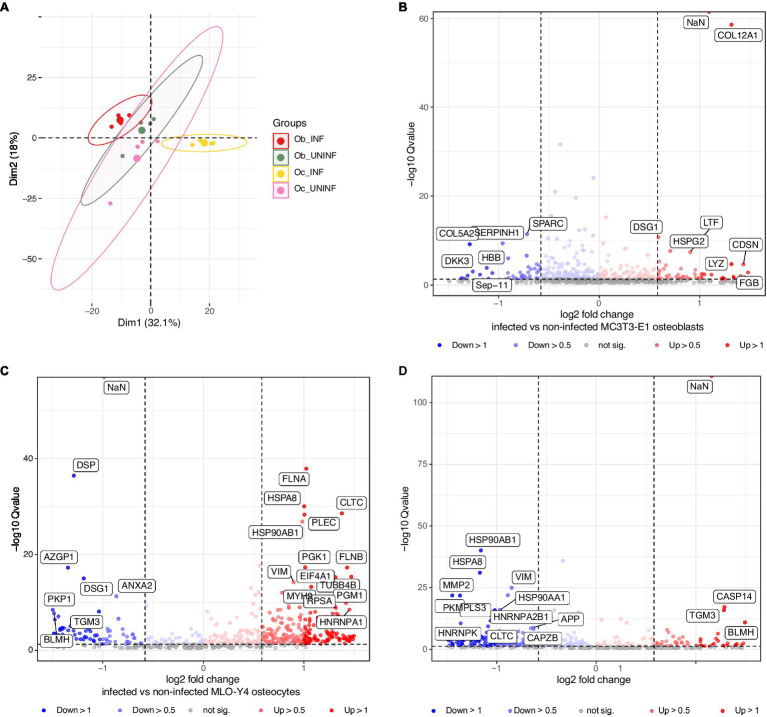
Proteomic analysis of osteoblasts and osteocytes infected with the *Staphylococcus aureus* LS1 strain. At 24 h post infection, the supernatants of infected and non-infected cells were collected and analyzed by mass spectrometry. **(A)** Principal component analysis (PCA) score plot comparing metabolic profiles of infected and non-infected osteoblasts and osteocytes. **(B–D)** Volcano plot (*q*-value vs. log_2_ fold change) for differentially expressed proteins: **(B)** Infected vs. non-infected osteoblasts. **(C)** Infected vs. non-infected osteocytes. **(D)** Infected osteoblasts vs. infected osteocytes. The *X* axis represents −log10Q value (values of *p* < 0.05); the *Y* axis represents log_2_ values of protein fold changes. See details in Supplementary Tables S3–5; *n* = 3.

To further characterize the response of osteoblasts and osteocytes to *S. aureus* infection, we performed proteomics analysis (Figure 5; Table 2; Supplementary Tables S3–5). Osteoblasts and osteocyte cell lines were infected with the *S. aureus* LS1 strain, and the supernatants were collected and analyzed by mass spectrometry. Principal component analysis (PCA) of the host cell proteins revealed that samples were clustered according to the treatment (infected vs. non-infected) rather than the cell type (osteoblasts vs. osteocytes; Figure 5A). In line with this observation, specific expression signatures were found for infected osteoblasts and osteocytes. Interestingly, a clear separation was seen between infected osteoblasts and osteocytes, while non-infected cells of osteoblast and osteocyte clustered closely together. These results suggest that osteoblast and osteocyte cell lines respond differently to *S. aureus* infection (Figure 5A).

**Table 2:** Antimicrobial proteins of infected osteoblasts vs. osteocytes (ob/oc).

Group	Protein groups	AVG Log2 Ratio	Qvalue	Protein description	Function
Q96FQ6	Q96FQ6	1.52	0.0008	Protein S100-A16	Antimicrobial peptide
P07339	P07339	1.58	0.0000	Cathepsin D	Antimicrobial peptide
P29508	P29508	1.83	0.0000	Serpin B3	Related to the expression of proteins S100/antimicrobial peptide
P61626	P61626	1.87	0.0001	Lysozyme C	Antimicrobial peptide
P06702	P06702	1.88	0.0000	Protein S100-A9	Antimicrobial peptide
Q96P63	Q96P63	1.94	0.0000	Serpin B12	Host defenses
O43240	O43240	1.95	0.0122	Kallikrein-10	Antimicrobial peptide
P81605	P81605	1.97	0.0000	Dermcidin	Antimicrobial peptide
P00441	P00441	2.02	0.0057	Superoxide dismutase [Cu-Zn]	Antimicrobial peptide
P31151	P31151	2.09	0.0008	Protein S100-A7	Antimicrobial peptide
Q8IW75	Q8IW75	2.27	0.0014	Serpin A12	Antimicrobial peptide
P05109	P05109	2.29	0.0003	Protein S100-A8	Antimicrobial peptide
Q9H1E1	Q9H1E1	2.39	0.0002	Ribonuclease 7	Antimicrobial peptide
Q6P4A8	Q6P4A8	2.45	0.0000	Phospholipase B-like 1	Antimicrobial peptide
O60911	O60911	2.74	0.0007	Cathepsin L2	Antimicrobial peptide
P02788	P02788	2.91	0.0000	Lactotransferrin	Antimicrobial peptide
O75635	O75635	3.17	0.0010	Serpin B7	Antimicrobial peptide
P31025	P31025	3.61	0.0009	Lipocalin-1	Antimicrobial peptide

Biological relevance of differentially expressed antimicrobial peptides between infected osteoblasts and osteocytes determined by proteomic analysis. Average of Log2ratios (AVG Log2-ratios) and *Q* values are indicated.

Next, metabolic pathway impact analysis was conducted in a pairwise manner to investigate the detailed pathway perturbations between and infected non-infected cells (Figures 5B,C; Supplementary Tables S3, S4). Comparison between infected vs. non-infected cells showed significant upregulation of pathways related to the induction of apoptosis and matrix degradation upon *S. aureus* infection [Supplementary Table S3: ob (osteoblasts) non-infected vs. ob infected and Supplementary Table S4: oc (osteocytes) non-infected vs. oc infected].

**Figure 6:**
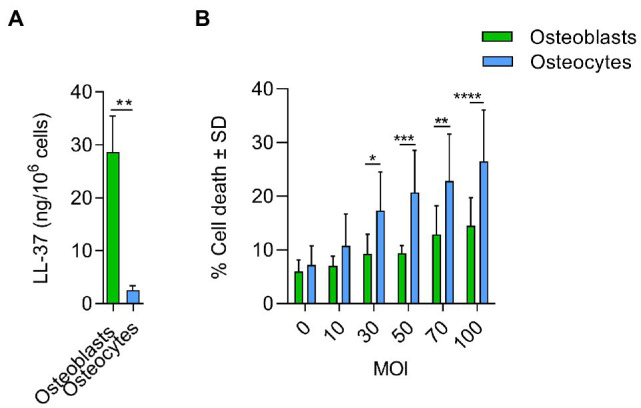
Antimicrobial response and cytotoxicity induced by *Staphylococcus aureus* in osteoblasts and osteocytes. **(A)** Secretion of LL-37 was measured by ELISA in osteoblasts and osteocytes cell free supernatants, 24 h after infection (ng/10^6^cells). Uninfected values were subtracted from infected values; *n* = 3. **(B)** % Cell death induced by *S. aureus* in osteoblasts and osteocytes at different doses (MOIs). Twenty-four hours post infection, cell death was measured by flow cytometry by staining cells with propidium iodine; *n* = 5. Statistical analysis was performed using the unpaired *t*-test and two-way ANOVA with Sidak’s multiple comparison test; *p < 0.05, **p < 0.01, ***p < 0.001, and ****p < 0.001. The bars and whiskers represent the means ±SD of different independent experiments.

Furthermore, the comparison between infected osteoblasts and osteocytes showed that the cells had differential metabolic profiles (Figure 5D). Proteins related to the extracellular matrix were significantly downregulated in osteoblasts compared to osteocytes. Interestingly, antimicrobial peptides were significantly upregulated in infected osteoblasts compared to osteocytes (Table 2). For validation of these results, secretion of LL-37, one of the main antimicrobial peptides, was measured in infected osteoblasts and osteocytes (Figure 6A). Secretion of LL-37 was significantly higher 24 h post infection with *S. aureus* LS1 in osteoblasts than in osteocytes. Taken together, our results suggest that infected osteocytes are not able to clear intracellular bacteria due to a reduced antimicrobial peptide production.

To test, whether the host cells can resist the infection, osteoblast and osteocyte cell lines were infected with increased doses of *S. aureus* (multiplicity of infection, MOIs) and the cytotoxic effect was measured (Figure 6B). Cytotoxicity was significantly higher in osteocytes than in osteoblasts at MOIs higher than 30, indicating that osteoblasts are better prepared to resist an infection than osteocytes.

### The Inflammatory Response Is More Pronounced in Infected Osteocytes Than in Osteoblasts

**Figure 7:**
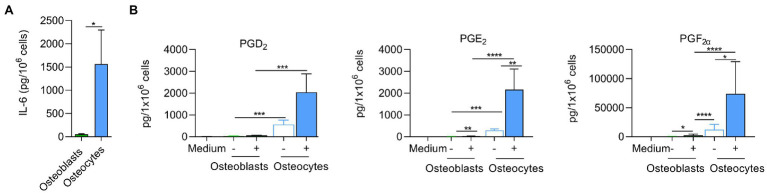
Inflammatory responses are more pronounced in infected osteocytes than in osteoblasts. **(A)** Infected cells were incubated for 24 h and IL-6 was measured by ELISA in cell free supernatants. Uninfected values were subtracted from infected values (pg/10^6^cells); *n* = 3. **(B)** Infected cells were incubated for 24 h. Next, extracted LMs were analyzed by UPLC-MS–MS (pg/10^6^cells); *n* = 6. Statistical analysis was performed using unpaired *t*-test with Welch’s correction: *p < 0.05, **p < 0.01, ***p < 0.001, and ****p < 0.0001. The bars and whiskers represent the means ±SEM of different independent experiments. −: control cells and +: infected cells.

*Staphylococcus aureus* infection triggers early cytokine secretion, such as TNF-α, IL-1β, and IL-6, which promotes the inflammatory response and recruits other immune cells to combat invading microorganisms (Brandt et al., 2018a). Osteoblasts and osteocytes were infected with *S. aureus* LS1, and the release of IL-6 was measured 24 h post infection. Interestingly, osteocytes released significantly more IL-6 than osteoblasts (Figure 7A).

To further investigate the inflammatory response, we analyzed the release of lipid mediators (LMs; Jimenez et al., 2005) at 24 h post infection in both bone cell lines (Figure 7B). Pro-inflammatory LMs such prostaglandins (PGs) and leukotrienes (LTs) are produced from arachidonic acid (AA, C20:4, ω-6) by cyclooxygenases (COXs) and 5-lipoxygenase (LOX), respectively (Jordan et al., 2020). These bioactive LMs are strongly involved in the inflammatory response and therefore may play a crucial role in the progression of prolonged osteomyelitis (Plotquin et al., 1991), bone destruction, and bacterial clearance. Bone cells were infected as described above, and the supernatants were collected 24 h post infection. The expression of LMs was measured by UPLC-MS–MS (Figure 7B; Supplementary Table S6). The infected osteocytes produced higher levels of pro-inflammatory COX-derived PGs than osteoblasts (Figure 7B; Supplementary Table S6). These results show that cyclooxygenase-mediated prostaglandins are upregulated in osteoblasts and osteocytes after exposure to pathogenic *S. aureus*, whereas osteocytes produce significantly higher amounts, especially for pro-inflammatory PGE_2_, suggesting stronger pro-inflammatory response *via* the COX pathway in these cells.

### Inhibition of TLR2 Does Not Affect Bacterial Persistence

**Figure 8:**
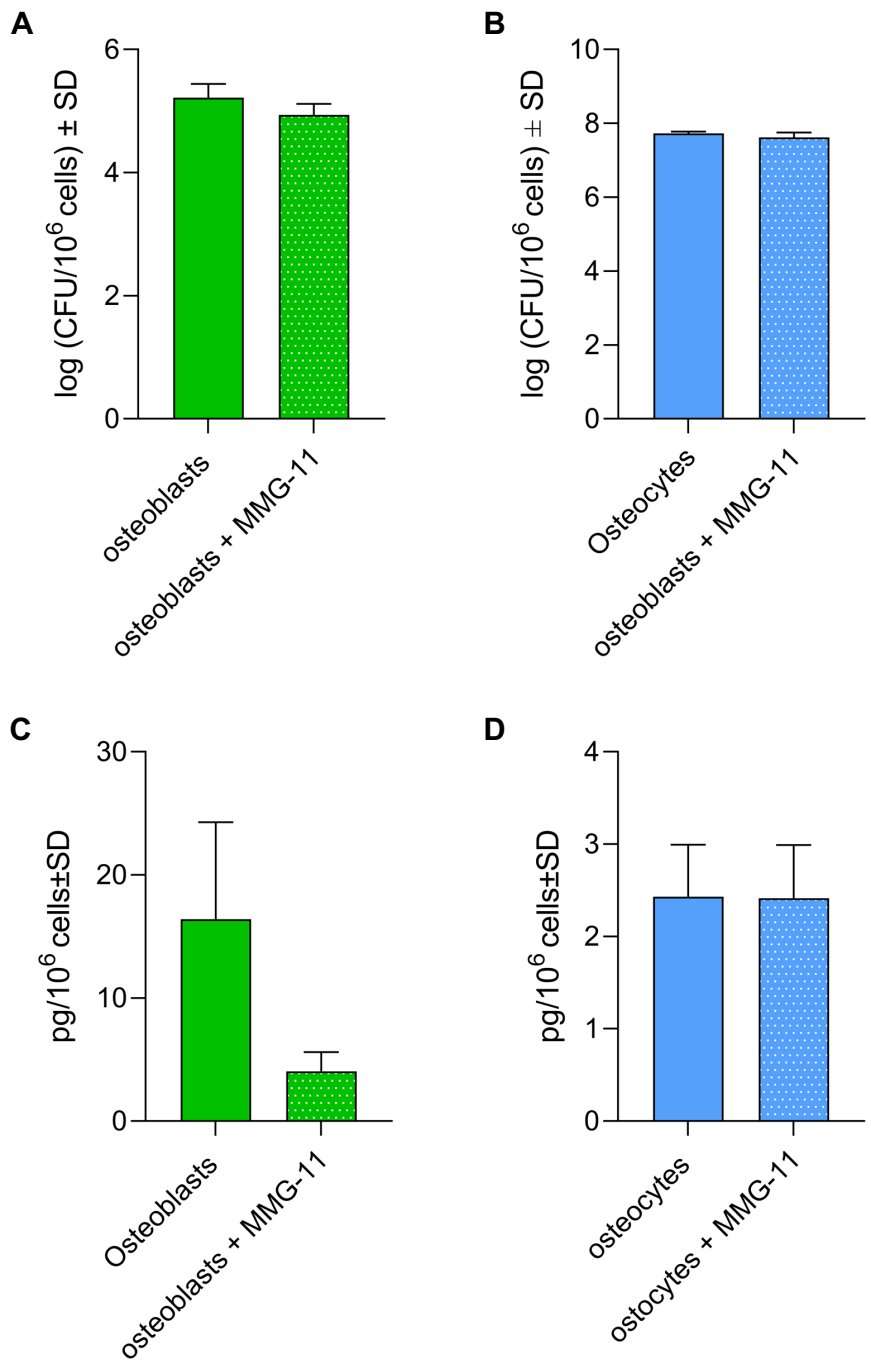
Inhibition of TLR2 does not affect bacterial persistence. **(A,B)** Osteoblasts **(A)** and osteocytes **(B)** were treated with 50 μM MMG-11 or not and infected with *S. aureus* LS1. Intracellular bacteria were quantified after 24 h post infection. **(C,D)** LL-37 was measured after 24 post infection from the supernatants from treated and non-treated osteoblasts **(C)** and osteocytes **(D)**. Statistical analysis was performed using unpaired *t*-test. The bars and whiskers represent the means ±SD of different independent experiments *n* = 3.

Taking in consideration that *S. aureus* was cleared faster from osteoblasts than osteocytes and the differences in TLR2 expression, we studied a possible link between persistence and TLR2. Osteoblasts and osteocytes were pre-treated or not with 50 μM of MMG-11 (TLR2 inhibitor) and infected with *S. aureus* LS1. Twenty-four hours post infection, intracellular bacteria were quantified by serial dilutions as described above and LL-37 was measured from all supernatants (Figure 8). Surprisingly, the inhibition of the TLR has not increased persistence; even a slight reduction has been observed (Figure 8A). Furthermore, the inhibition of TLR2 did not affect the intracellular bacterial load in osteocytes (Figure 8B). However, almost significant differences in LL-37 release were observed in TLR2-inhibited osteoblasts compared to non-treated cells (*p* = 0.055; Figure 8C). Interestingly, the amount of secreted LL-37 was much higher in osteoblasts when compared to osteocytes. The amount of LL-37 did not differed between treated and untreated osteocytes (Figure 8D). Taken together, our results suggested that the differences in TLR2 expression were not directly linked to intracellular persistence of *S. aureus* within osteocytes.

## Discussion

*Staphylococcus aureus* osteomyelitis is a severe and destructive infection that can develop into a chronic course. Recent findings have shown that *S. aureus* can invade bone cells during chronic osteomyelitis providing a niche for bacterial persistence (Yang et al., 2018; Masters et al., 2021). However, the characteristics of bone cells that facilitate staphylococcal persistence have been poorly investigated so far.

In the present study, we focused on osteoblasts and osteocytes, as main non-phagocytic cells in bone tissue. We show that osteocytes have a dual role during *S. aureus* infection, that is, they secrete several mediators that foster the inflammatory response but fail to eliminate intracellular *S. aureus*. In this way they represent a safe niche for intracellular bacterial persistence. In contrast, osteoblasts can efficiently eliminate intracellular bacteria and contribute partially to the inflammatory response.

In this study, bone cell infections were performed in *in vitro* mono- and co-culture models for 7 days. No differences were evident between osteoblasts and osteocytes directly after host cell invasion. A possible mechanism for *S. aureus* invasion into osteoblasts and osteocytes might be the recognition of α5β1 integrins on host cells through fibronectin binding proteins (FnBPA and FnBPB; Niemann et al., 2021). However, further experiments should be performed to investigate this pathway in the present model.

Following internalization, the bacteria can evade cell death *via* persistence within vacuoles, endosomal escape, or by preventing phagolysosomal fusion (Moldovan and Fraunholz, 2019; Siegmund et al., 2021). Intracellular persistence was investigated in cell culture of both cell types up to 7 days. Higher intracellular bacteria counts were found in osteocytes compared to osteoblasts. These results are in line with other studies that propose osteocytes as a niche for staphylococcal persistence (Muthukrishnan et al., 2019; Gunn et al., 2021). Staphylococcal persistence is related to a switch to a slow-growing, metabolically inactive phenotype called small colony variants (SCVs; Sendi et al., 2006; Tuchscherr et al., 2011). SCVs were detectable within osteoblasts and osteocytes as previously described (Yang et al., 2018). Higher SCV formation was found in osteocytes than in osteoblasts in mono- and co-culture models. These results suggest that the osteocyte intracellular environment favors SCV formation, contributing to the chronicity of the infection. Culturing osteoblasts with conditioned medium obtained from infected bone cells (osteocytes or osteoblasts) primed the osteoblasts resulting in significantly more killed intracellular bacteria compared to the control culture. However, no differences in bacterial clearance were found when osteocytes were cultured with infected conditioned medium and control conditioned medium. After infection, osteoblasts secrete cytokines, chemokines, enzymes and antimicrobial peptides (Josse et al., 2015; Brandt et al., 2018b). These signals are recognized by receptors such as toll-like receptors present on the membrane of other cells to activate antimicrobial response. Our results suggest that osteoblasts recognized molecules present in conditioned media and trigger the killing of intracellular bacteria. In contrast, osteocytes were not able to recognize these signals and failed to clear the intracellular bacteria. The lack of intracellular bacterial killing can be related to (1) a deficiency or absence of host receptors that recognize pathogens and/or (2) a deficient induction of host defenses (Brandt et al., 2018b).

Toll-like receptors (TLRs) play a central role in innate immunity by mediating the recognition of pathogen-associated microbial patterns (PAMPs; Brandt et al., 2018b). As the recognition of *S. aureus* by TLR2 is linked with the host immune response activation (De Oliviera Nascimento et al., 2012), we analyzed the presence of this receptor on both cell lines by flow cytometry. We demonstrated that the expression of TLR2 was higher in infected osteoblasts than in uninfected ones. However, non- and infected osteocytes expressed similar amounts of TLR2. Since the activation of antimicrobial peptides is linked to TLR2 (Varoga et al., 2008a), we thought that there can be a link between the low expression of this receptor and the persistence of *S. aureus* within osteocytes. However, inhibition of TLR2 receptor in osteoblasts did not increase the bacterial persistence. These results are in contrast to our assumption. Of note, a previous study performed in monocytes found the opposite effect of what we expected based on our data: the persistence of *S. aureus* was reduced in knock out cells for TLR2 (Musilova et al., 2019). However, the expression of LL-37 was reduced after TLR2 inhibition in osteoblasts. Therefore, future studies will be necessary to find out which pathway is involved in the expression of antimicrobial peptides in bone cells, since the expression of antimicrobial peptides is influenced by other systems (Askarian et al., 2018).

Following bacterial stimulation, a signaling cascade is initiated that triggers the nuclear translocation of nuclear factor-κB (NF-κB). This nuclear factor modulates the transcription of the genes that participate in the antimicrobial response and in inflammatory cytokine production (Varoga et al., 2008a,b; De Oliviera Nascimento et al., 2012). The host antimicrobial response involves the production of antibacterial weapons against pathogens such as antimicrobial peptides (Varoga et al., 2008b; Brandt et al., 2018a). Due to the differences in persistence in both bone cells, we expected a reduced antimicrobial response in osteocytes. In fact, proteomic analysis of the supernatants of both bone cell lines revealed a significant downregulation of antimicrobial peptides in osteocytes. Moreover, these results were confirmed by measurement of a higher level of LL-37 in the supernatant of infected osteoblasts compared to osteocytes.

According to proteomic analysis, we conclude that the survival of *S. aureus* within osteocytes is promoted by the lack of an efficient antimicrobial response. In fact, these results suggest that, compared to osteoblasts, osteocytes are more susceptible to *S. aureus* infection. By testing increasing doses of *S. aureus* on both bone cells, we observed highly significant cytotoxic effects on osteocytes at very high doses. However, no differences were observed with low doses. By contrast, a recent publication has shown that osteoblasts were more susceptible to *S. aureus* infection than osteocytes (Gunn et al., 2021). This possible discrepancy may be related to the staphylococcal strains and cells used in both studies. In general, the cell death induced by *S. aureus* in osteoblasts and osteocytes might contribute to the destructive bone loss that can be observed in chronic forms of osteomyelitis (Yang et al., 2018). Of note, the intracellular environment of osteoblasts and osteocytes may differ and may influence the bacterial regulation of intracellular bacteria. The effect of intracellular environment on bacterial virulence regulation may affect as well the outcome of the infection. Further proteomic analysis on intracellular *S. aureus* within both bone cells is needed to investigate this point.

Immune cells are attracted to the infection due to cytokines and LMs (Sadik and Luster, 2012). To determine the role of both bone cells in the recruitment of immune cells, we investigated the release of IL-6 and LMs in the supernatant from infected cells. Interestingly, a highly significant secretion of IL-6 was found by osteocytes infected with *S. aureus*. Furthermore, the pro-inflammatory LMs related to COX were secreted by infected osteocytes but not by osteoblasts. These results are in line with a previous study in which the expression of COX was measured at high concentrations in osteocytes in samples from patients who suffered from osteoarthritis (Tu et al., 2019).

Of note, osteoblasts and osteocytes released prostaglandins (PGs) following a *S. aureus* infection, but significantly higher secretion of PGE_2_ was found in osteocytes than in osteoblasts. PGE_2_ can modulate several pathways that can either have adverse or beneficial effects on the ability of the immune system to fend off pathogens (Kalinski, 2012). High production of PGE_2_ was proposed as a mechanism to prevent osteocytes apoptosis *via* Wnt pathway. According to our results, *S. aureus* may trigger a protective effect on osteocytes through PGE_2_ (Bonewald and Johnson, 2008) to prevent destruction of these host cells and promote its intracellular persistence. Further experiments are necessary to determine the relationship between PGE_2_, apoptosis and *S. aureus* infection.

The data gained from the different experimental approaches used in this study indicate dedicated defense mechanisms of osteoblasts and osteocytes against *S. aureus*. Further studies are necessary now, to elucidate these in more detail. A limitation of these studies is the use of cell lines of osteocytes and osteoblasts which might respond differently compared to primary bone cells, and intense data comparisons are necessary to define physiologically relevant effects. This limitation is difficult to overcome at the moment due to the lack of efficient protocols to isolate primary osteocytes in high amounts. However, the used MLO-Y4 cell line is a very established cell line for osteocyte research. The MLO-Y4 cells are different to primary osteocytes (*in vitro/in vivo*) as they do not express the osteocyte marker Sclerostin and FGF23 (Zhang et al., 2019). Further cell lines have been developed, such as the pre-osteocytes ML0-A5, pre- to late-osteocytes IDG-SW3 and mature osteocytes Ocy 454. They all express sclerostin, but they also have limitations, as they are also modified proliferating cell lines (Zhang et al., 2019). MLO-Y4 cells build dendritic extensions connecting them to neighboring cells in 2D culture (Wu et al., 2013) as seen *in vivo*. For the osteoblast experiments the well-established cell line MC3T3-E1 subclone 4 was used. As described by Hwang et al., the MC3T3 sub clones show differences between each other and also compared to primary osteoblasts on a transcriptional level and regarding mineralization capacity (Hwang and Horton, 2019). However, primary cells would also show a high variability making the use of cells from different donors necessary. The isolation of human osteocyte from human trabecular bone has been described (Prideaux et al., 2016); but the number of harvested cells is very low and not sufficient to be used in larger experimental series. Recently, a new model of a human osteocyte cell line with SaO-2 cells was described for studying *S. aureus* persistence (Gunn et al., 2021). In this study, the SaO-2 cells were cultivated for 28 days to obtain osteocytes-like cells. However, these cells lack the expression of E11, one of the osteocytes markers, and express a phenotype more related to the transition from osteoblasts to osteocytes (Prideaux et al., 2014; Gunn et al., 2021). To mimic the human *in vivo* situation best, 3D cultures systems of human primary osteocytes in combination with osteoblast and osteoclast have been developed (Bernhardt et al., 2020) and these systems might be suitable for further investigations of bacterial persistence in bone cells.

In addition, anatomical and morphological differences between the *in vivo* and *in vitro* situation may play a role in the clearance of *S. aureus* by bone cells. Osteocytes cell line MLO-Y4 expresses a similar dendritic phenotype as primary osteocytes. However, MLO-Y4 cells under *in vitro* conditions used in this study grow in 2D. Even though, 3D *in vivo* osteocytes might have a larger surface, the contact area to *S. aureus* might be smaller, because the cells are embedded in the bone matrix. *In vitro*, the entire cell is exposed to the bacteria and therefore more bacteria might invade the cells. Moreover, osteocytes are relatively inaccessible to immune cells *in vivo* due to their localization within the matrix (Prideaux et al., 2014; Gunn et al., 2021). Contrary, osteoblasts are located on the bone surface and exposed to bacteria and immune cells during infection (Gunn et al., 2021). These characteristics may affect the entry of *S. aureus* and its clearance from osteocytes between the *in vivo* and *in vitro* scenario.

## Conclusion

Taken together, our findings demonstrate the ability of *S. aureus* to survive and persist within osteocytes due to poor intracellular bacterial clearance. We revealed a controversial role of osteocytes versus osteoblasts during infection, with osteocytes representing the main promotor of the inflammatory response that might mediate bone destruction in chronic osteomyelitis and at the same time being the main reservoir for intracellular *S. aureus*.

## Data Availability Statement

The mass spectrometry proteomics data have been deposited to the ProteomeXchange Consortium via the PRIDE partner repository with the dataset identifier PRIDE: PXD029440.

## Author Contributions

MG-M: performed all *in vitro* experiments, analyzed the data, designed and generated the figures, and contributed to the writing of the manuscript. PJ and KG: planned, performed, and analyzed the *in vitro* measurement of TLR2 and lipid mediators. TD: performed the proteomic analysis and generated the related figures. CF and MV: contributed to the cell infection experiments. AS: contributed to the discussion of the results. AI and BW: contributed to the discussion and writing of the manuscript. OW and BL: contributed to the discussion of the results and writing of the manuscript. LT: planned, supervised, and designed the experiments, discussed the results, and wrote the manuscript. All authors contributed to the article and approved the submitted version.

## Funding

This work was supported by grants from the MESINFLAME (BMBF grant no. 01EC1901B: BL and LT), German Research Foundation (DFG; CRC1149, project ID 251293561, and C01 INST 40/491-2: AI and AS), the Deutsche Forschungsgemeinschaft (DFG, German Research Foundation, project-ID 239748522, SFB 1127 ChemBioSys, and project A04: OW), project-ID 316213987–SFB 1278 PolyTarget (projects A04: OW; D02: BL), and project-ID 210879364–CRC/TRR124 FungiNet (project A7N: OW).

## Conflict of Interest

The authors declare that the research was conducted in the absence of any commercial or financial relationships that could be construed as a potential conflict of interest.

## Publisher’s Note

All claims expressed in this article are solely those of the authors and do not necessarily represent those of their affiliated organizations, or those of the publisher, the editors and the reviewers. Any product that may be evaluated in this article, or claim that may be made by its manufacturer, is not guaranteed or endorsed by the publisher.

## Footnotes

^1^ http://www.ebi.ac.uk/pride

## References

[ref1] Ahmed S. Meghji S. Williams R. J. Henderson B. Brock J. H. Nair S. P. (2001). *Staphylococcus aureus* fibronectin binding proteins are essential for internalization by osteoblasts but do not account for differences in intracellular levels of bacteria. Infect. Immun. 69, 2872–2877. doi: 10.1128/IAI.69.5.2872-2877.2001, PMID: 11292701 PMC98237

[ref2] Alder K. D. Lee I. Munger A. M. Kwon H. K. Morris M. T. Cahill S. V.. (2020). Intracellular *Staphylococcus aureus* in bone and joint infections: a mechanism of disease recurrence, inflammation, and bone and cartilage destruction. Bone 141:115568. doi: 10.1016/j.bone.2020.115568, PMID: 32745687

[ref3] Askarian F. Wagner T. Johannessen M. Nizet V. (2018). *Staphylococcus aureus* modulation of innate immune responses through toll-like (TLR), (NOD)-like (NLR) and C-type lectin (CLR) receptors. FEMS Microbiol. Rev. 42, 656–671. doi: 10.1093/femsre/fuy025, PMID: 29893825 PMC6098222

[ref4] Bernhardt A. Österreich V. Gelinsky M. (2020). Three-dimensional co-culture of primary human osteocytes and mature human osteoclasts in collagen gels. Tissue Eng. Part A 26, 647–655. doi: 10.1089/ten.tea.2019.0085, PMID: 31774039

[ref5] Bonewald L. F. Johnson M. L. (2008). Osteocytes, mechanosensing and Wnt signaling. Bone 42, 606–615. doi: 10.1016/j.bone.2007.12.224, PMID: 18280232 PMC2349095

[ref6] Bongiorno D. Musso N. Caruso G. Lazzaro L. M. Caraci F. Stefani S.. (2021). *Staphylococcus aureus* ST228 and ST239 as models for expression studies of diverse markers during osteoblast infection and persistence. Microbiology 10:e1178. doi: 10.1002/mbo3.1178, PMID: 33970534 PMC8087985

[ref7] Brandt S. L. Putnam N. E. Cassat J. E. Serezani C. H. (2018a). Innate immunity to <em>*Staphylococcus aureus*</em>: evolving paradigms in soft tissue and invasive infections. J. Immunol. 200, 3871–3880. doi: 10.4049/jimmunol.1701574, PMID: 29866769 PMC6028009

[ref8] Brandt S. L. Putnam N. E. Cassat J. E. Serezani C. H. (2018b). Innate immunity to *Staphylococcus aureus*: evolving paradigms in soft tissue and invasive infections. J. Immunol. 200, 3871–3880. doi: 10.4049/jimmunol.1701574, PMID: 29866769 PMC6028009

[ref9] Colas R. A. Shinohara M. Dalli J. Chiang N. Serhan C. N. (2014). Identification and signature profiles for pro-resolving and inflammatory lipid mediators in human tissue. Am. J. Phys. Cell Physiol. 307, C39–C54. doi: 10.1152/ajpcell.00024.2014, PMID: 24696140 PMC4080182

[ref10] De Jong N. W. M. Van Kessel K. P. M. Van Strijp J. A. G. (2019). Immune evasion by *Staphylococcus aureus*. Microbiol. Spectr. 7, 1–27. doi: 10.1128/microbiolspec.GPP3-0061-2019, PMID: 30927347 PMC11590434

[ref11] De Mesy Bentley K. L. Macdonald A. Schwarz E. M. Oh I. (2018). Chronic osteomyelitis with *Staphylococcus aureus* deformation in submicron Canaliculi of osteocytes: a case report. JBJS Case Connect 8:e8. doi: 10.2106/JBJS.CC.17.00154, PMID: 29443819 PMC6681818

[ref12] De Oliviera Nascimento L. Massari P. Wetzler L. (2012). The role of TLR2 in infection and immunity. Front. Immunol. 3:79. doi: 10.3389/fimmu.2012.00079, PMID: 22566960 PMC3342043

[ref13] Fritz J. M. Mcdonald J. R. (2008). Osteomyelitis: approach to diagnosis and treatment. Phys. Sportsmed. 36:nihpa116823. doi: 10.3810/psm.2008.12.11, PMID: 19652694 PMC2696389

[ref14] Garcia L. G. Lemaire S. Kahl B. C. Becker K. Proctor R. A. Denis O.. (2013). Antibiotic activity against small-colony variants of *Staphylococcus aureus*: review of in vitro, animal and clinical data. J. Antimicrob. Chemother. 68, 1455–1464. doi: 10.1093/jac/dkt072, PMID: 23485724

[ref15] Gunn N. J. Zelmer A. R. Kidd S. P. Solomon L. B. Roscioli E. Yang D.. (2021). A human osteocyte cell line model for studying *Staphylococcus aureus* persistence in osteomyelitis. Front. Cell. Infect. Microbiol. 11:781022. doi: 10.3389/fcimb.2021.781022, PMID: 34805001 PMC8597899

[ref16] Horn J. Stelzner K. Rudel T. Fraunholz M. (2018). Inside job: *Staphylococcus aureus* host-pathogen interactions. Int. J. Med. Microbiol. 308, 607–624. doi: 10.1016/j.ijmm.2017.11.009, PMID: 29217333

[ref17] Horsburgh M. J. Aish J. L. White I. J. Shaw L. Lithgow J. K. Foster S. J. (2002). sigmaB modulates virulence determinant expression and stress resistance: characterization of a functional rsbU strain derived from Staphylococcus aureus 8325-4. J. Bacteriol. 184, 5457–5467. doi: 10.1128/JB.184.19.5457-5467.2002, PMID: 12218034 PMC135357

[ref18] Hwang P. W. Horton J. A. (2019). Variable osteogenic performance of MC3T3-E1 subclones impacts their utility as models of osteoblast biology. Sci. Rep. 9:8299. doi: 10.1038/s41598-019-44575-8, PMID: 31165768 PMC6549152

[ref19] Ji Z. Su J. Hou Y. Yao Z. Yu B. Zhang X. (2020). EGFR/FAK and c-Src signalling pathways mediate the internalisation of *Staphylococcus aureus* by osteoblasts. Cell. Microbiol. 22:e13240. doi: 10.1111/cmi.13240, PMID: 32584493

[ref20] Jimenez R. Belcher E. Sriskandan S. Lucas R. Mcmaster S. Vojnovic I.. (2005). Role of toll-like receptors 2 and 4 in the induction of cyclooxygenase-2 in vascular smooth muscle. Proc. Natl. Acad. Sci. U. S. A. 102, 4637–4642. doi: 10.1073/pnas.0407655101, PMID: 15755814 PMC555494

[ref21] Joo H. S. Otto M. (2015). Mechanisms of resistance to antimicrobial peptides in *Staphylococci*. Biochim. Biophys. Acta 1848, 3055–3061. doi: 10.1016/j.bbamem.2015.02.009, PMID: 25701233 PMC4539291

[ref22] Jordan P. M. Gerstmeier J. Pace S. Bilancia R. Rao Z. Börner F.. (2020). *Staphylococcus aureus*-derived α-Hemolysin evokes generation of specialized pro-resolving mediators promoting inflammation resolution. Cell Rep. 33:108247. doi: 10.1016/j.celrep.2020.108247, PMID: 33053344 PMC7729929

[ref23] Josse J. Velard F. Gangloff S. C. (2015). *Staphylococcus aureus* vs. osteoblast: relationship and consequences in osteomyelitis. Front. Cell. Infect. Microbiol. 5:85. doi: 10.3389/fcimb.2015.00085, PMID: 26636047 PMC4660271

[ref24] Kahl B. C. Becker K. Löffler B. (2016). Clinical significance and pathogenesis of staphylococcal small Colony variants in persistent infections. Clin. Microbiol. Rev. 29, 401–427. doi: 10.1128/CMR.00069-15, PMID: 26960941 PMC4786882

[ref25] Kalinka J. Hachmeister M. Geraci J. Sordelli D. Hansen U. Niemann S.. (2014). *Staphylococcus aureus* isolates from chronic osteomyelitis are characterized by high host cell invasion and intracellular adaptation, but still induce inflammation. Int. J. Med. Microbiol. 304, 1038–1049. doi: 10.1016/j.ijmm.2014.07.013, PMID: 25129555

[ref26] Kalinski P. (2012). Regulation of immune responses by prostaglandin E2. J. Immunol. 188, 21–28. doi: 10.4049/jimmunol.1101029, PMID: 22187483 PMC3249979

[ref27] Kavanagh N. Ryan E. J. Widaa A. Sexton G. Fennell J. O'Rourke S.. (2018). Staphylococcal osteomyelitis: disease progression, treatment challenges, and future directions. Clin. Microbiol. Rev. 31, e00084–e00117. doi: 10.1128/CMR.00084-17, PMID: 29444953 PMC5967688

[ref28] Krauss J. L. Roper P. M. Ballard A. Shih C. C. Fitzpatrick J. A. J. Cassat J. E.. (2019). *Staphylococcus aureus* infects osteoclasts and replicates intracellularly. MBio 10, e02447–e02519. doi: 10.1128/mBio.02447-19, PMID: 31615966 PMC6794488

[ref29] Marro F. C. Abad L. Blocker A. J. Laurent F. Josse J. Valour F. (2021). In vitro antibiotic activity against intraosteoblastic *Staphylococcus aureus*: a narrative review of the literature. J. Antimicrob. Chemother. 76, 3091–3102. doi: 10.1093/jac/dkab301, PMID: 34459881 PMC8598303

[ref30] Masters E. A. Muthukrishnan G. Ho L. Gill A. L. De Mesy Bentley K. L. Galloway C. A.. (2021). *Staphylococcus aureus* cell wall biosynthesis modulates bone invasion and osteomyelitis pathogenesis. Front. Microbiol. 12:723498. doi: 10.3389/fmicb.2021.723498, PMID: 34484165 PMC8415456

[ref31] Moldovan A. Fraunholz M. J. (2019). In or out: Phagosomal escape of *Staphylococcus aureus*. Cell. Microbiol. 21:e12997. doi: 10.1111/cmi.12997 30576050

[ref32] Musilova J. Mulcahy M. E. Kuijk M. M. Mcloughlin R. M. Bowie A. G. (2019). Toll-like receptor 2-dependent endosomal signaling by *Staphylococcus aureus* in monocytes induces type I interferon and promotes intracellular survival. J. Biol. Chem. 294, 17031–17042. doi: 10.1074/jbc.RA119.009302, PMID: 31558608 PMC6851302

[ref33] Muthukrishnan G. Masters E. A. Daiss J. L. Schwarz E. M. (2019). Mechanisms of immune evasion and bone tissue colonization that make *Staphylococcus aureus* the primary pathogen in osteomyelitis. Curr. Osteoporos. Rep. 17, 395–404. doi: 10.1007/s11914-019-00548-4, PMID: 31721069 PMC7344867

[ref34] Niemann S. Nguyen M. T. Eble J. A. Chasan A. I. Mrakovcic M. Böttcher R. T.. (2021). More is not always better-the double-headed role of fibronectin in *Staphylococcus aureus* host cell invasion. MBio 12:e0106221. doi: 10.1128/mBio.01062-21, PMID: 34663090 PMC8524341

[ref35] Pfaffl M. W. (2001). A new mathematical model for relative quantification in real-time RT-PCR. Nucleic Acids Res. 29:e45. doi: 10.1093/nar/29.9.e45, PMID: 11328886 PMC55695

[ref36] Plotquin D. Dekel S. Katz S. Danon A. (1991). Prostaglandin release by normal and osteomyelitic human bones. Prostaglandins Leukot. Essent. Fat. Acids 43, 13–15. doi: 10.1016/0952-3278(91)90126-P, PMID: 1881938

[ref37] Prideaux M. Schutz C. Wijenayaka A. R. Findlay D. M. Campbell D. G. Solomon L. B.. (2016). Isolation of osteocytes from human trabecular bone. Bone 88, 64–72. doi: 10.1016/j.bone.2016.04.017, PMID: 27109824

[ref38] Prideaux M. Wijenayaka A. R. Kumarasinghe D. D. Ormsby R. T. Evdokiou A. Findlay D. M.. (2014). SaOS2 osteosarcoma cells as an in vitro model for studying the transition of human osteoblasts to osteocytes. Calcif. Tissue Int. 95, 183–193. doi: 10.1007/s00223-014-9879-y, PMID: 24916279

[ref39] Prince A. Wong Fok Lung T. (2020). Consequences of metabolic interactions during *Staphylococcus aureus* infection. Toxins 12:581. doi: 10.3390/toxins12090581, PMID: 32917040 PMC7551354

[ref40] Proctor R. (2019). Respiration and small Colony variants of *Staphylococcus aureus*. Microbiol. Spectr. 7, 1–15. doi: 10.1128/microbiolspec.GPP3-0069-2019, PMID: 31198131 PMC11257146

[ref41] Proctor R. A. Von Eiff C. Kahl B. C. Becker K. Mcnamara P. Herrmann M.. (2006). Small colony variants: a pathogenic form of bacteria that facilitates persistent and recurrent infections. Nat. Rev. Microbiol. 4, 295–305. doi: 10.1038/nrmicro1384, PMID: 16541137

[ref42] Robling A. G. Bonewald L. F. (2020). The osteocyte: new insights. Annu. Rev. Physiol. 82, 485–506. doi: 10.1146/annurev-physiol-021119-034332, PMID: 32040934 PMC8274561

[ref43] Sadik C. D. Luster A. D. (2012). Lipid-cytokine-chemokine cascades orchestrate leukocyte recruitment in inflammation. J. Leukoc. Biol. 91, 207–215. doi: 10.1189/jlb.0811402, PMID: 22058421 PMC3290425

[ref44] Sendi P. Rohrbach M. Graber P. Frei R. Ochsner P. E. Zimmerli W. (2006). *Staphylococcus aureus* small Colony variants in prosthetic joint infection. Clin. Infect. Dis. 43, 961–967. doi: 10.1086/507633, PMID: 16983605

[ref45] Siegmund A. Afzal M. A. Tetzlaff F. Keinhörster D. Gratani F. Paprotka K.. (2021). Intracellular persistence of *Staphylococcus aureus* in endothelial cells is promoted by the absence of phenol-soluble modulins. Virulence 12, 1186–1198. doi: 10.1080/21505594.2021.1910455, PMID: 33843450 PMC8043190

[ref46] Thompson S. Townsend R. (2011). Pharmacological agents for soft tissue and bone infected with MRSA: which agent and for how long? Injury 42, S7–S10. doi: 10.1016/S0020-1383(11)70126-7, PMID: 22196911

[ref47] Traber K. E. Lee E. Benson S. Corrigan R. Cantera M. Shopsin B.. (2008). Agr function in clinical *Staphylococcus aureus* isolates. Microbiology 154, 2265–2274. doi: 10.1099/mic.0.2007/011874-0, PMID: 18667559 PMC4904715

[ref48] Tu M. Yang M. Yu N. Zhen G. Wan M. Liu W.. (2019). Inhibition of cyclooxygenase-2 activity in subchondral bone modifies a subtype of osteoarthritis. Bone Res. 7:29. doi: 10.1038/s41413-019-0071-x, PMID: 31666999 PMC6804921

[ref49] Tuchscherr L. Bischoff M. Lattar S. M. Noto Llana M. Pförtner H. Niemann S.. (2015). Sigma factor SigB is crucial to mediate *Staphylococcus aureus* adaptation during chronic infections. PLoS Pathog. 11:e1004870. doi: 10.1371/journal.ppat.1004870, PMID: 25923704 PMC4414502

[ref50] Tuchscherr L. Heitmann V. Hussain M. Viemann D. Roth J. Von Eiff C.. (2010). *Staphylococcus aureus* small-colony variants are adapted phenotypes for intracellular persistence. J. Infect. Dis. 202, 1031–1040. doi: 10.1086/656047, PMID: 20715929

[ref51] Tuchscherr L. Kreis C. A. Hoerr V. Flint L. Hachmeister M. Geraci J.. (2016). *Staphylococcus aureus* develops increased resistance to antibiotics by forming dynamic small colony variants during chronic osteomyelitis. J. Antimicrob. Chemother. 71, 438–448. doi: 10.1093/jac/dkv371, PMID: 26589581

[ref52] Tuchscherr L. Löffler B. Proctor R. A. (2020). Persistence of *Staphylococcus aureus*: multiple metabolic pathways impact the expression of virulence factors in small-Colony variants (SCVs). Front. Microbiol. 11:1028. doi: 10.3389/fmicb.2020.01028, PMID: 32508801 PMC7253646

[ref53] Tuchscherr L. Medina E. Hussain M. Völker W. Heitmann V. Niemann S.. (2011). *Staphylococcus aureus* phenotype switching: an effective bacterial strategy to escape host immune response and establish a chronic infection. EMBO Mol. Med. 3, 129–141. doi: 10.1002/emmm.201000115, PMID: 21268281 PMC3395110

[ref54] Varoga D. Tohidnezhad M. Paulsen F. Wruck C. J. Brandenburg L. Mentlein R.. (2008a). The role of human beta-defensin-2 in bone. J. Anat. 213, 749–757. doi: 10.1111/j.1469-7580.2008.00992.x, PMID: 19094191 PMC2666144

[ref55] Varoga D. Wruck C. J. Tohidnezhad M. Brandenburg L. Paulsen F. Mentlein R.. (2008b). Osteoblasts participate in the innate immunity of the bone by producing human beta defensin-3. Histochem. Cell Biol. 131, 207–218. doi: 10.1007/s00418-008-0522-8, PMID: 18925411

[ref56] Werner M. (2019). ECHAM5-wiso simulation data—present-day, mid-Holocene, and last glacial maximum. PANGAEA.

[ref57] Werz O. Gerstmeier J. Libreros S. De La Rosa X. Werner M. Norris P. C.. (2018). Human macrophages differentially produce specific resolvin or leukotriene signals that depend on bacterial pathogenicity. Nat. Commun. 9:59. doi: 10.1038/s41467-017-02538-5, PMID: 29302056 PMC5754355

[ref58] Wickersham M. Wachtel S. Wong Fok Lung T. Soong G. Jacquet R. Richardson A.. (2017). Metabolic stress drives keratinocyte defenses against *Staphylococcus aureus* infection. Cell Rep. 18, 2742–2751. doi: 10.1016/j.celrep.2017.02.055, PMID: 28297676 PMC6799992

[ref59] Wong Fok Lung T. Monk I. R. Acker K. P. Mu A. Wang N. Riquelme S. A.. (2020). *Staphylococcus aureus* small colony variants impair host immunity by activating host cell glycolysis and inducing necroptosis. Nat. Microbiol. 5, 141–153. doi: 10.1038/s41564-019-0597-0, PMID: 31686028 PMC10184863

[ref60] Wu D. Schaffler M. B. Weinbaum S. Spray D. C. (2013). Matrix-dependent adhesion mediates network responses to physiological stimulation of the osteocyte cell process. Proc. Natl. Acad. Sci. U. S. A. 110, 12096–12101. doi: 10.1073/pnas.1310003110, PMID: 23818616 PMC3718158

[ref61] Yang D. Wijenayaka A. R. Solomon L. B. Pederson S. M. Findlay D. M. Kidd S. P.. (2018). Novel insights into *Staphylococcus aureus* deep bone infections: the involvement of osteocytes. MBio 9, e00415–e00418. doi: 10.1128/mBio.00415-18 29691335 PMC5915738

[ref62] Zhang C. Bakker A. D. Klein-Nulend J. Bravenboer N. (2019). Studies on osteocytes in their 3D native matrix versus 2D in vitro models. Curr. Osteoporos. Rep. 17, 207–216. doi: 10.1007/s11914-019-00521-1, PMID: 31240566 PMC6647862

