# Supplementary material for: Osteocytes Serve as a Reservoir for Intracellular Persisting Staphylococcus aureus Due to the Lack of Defense Mechanisms
Source: Front Microbiol. 2022 Jul 22;13:937466. doi: 10.3389/fmicb.2022.937466 (PMC9355688; doi:10.3389/fmicb.2022.937466)
Supplement: Supplementary file 1 [file Data_Sheet_1.zip › Supplementary Material/SM Figures 1 - 6, Tables 1, 2 and 6.pptx]

## Slide 1
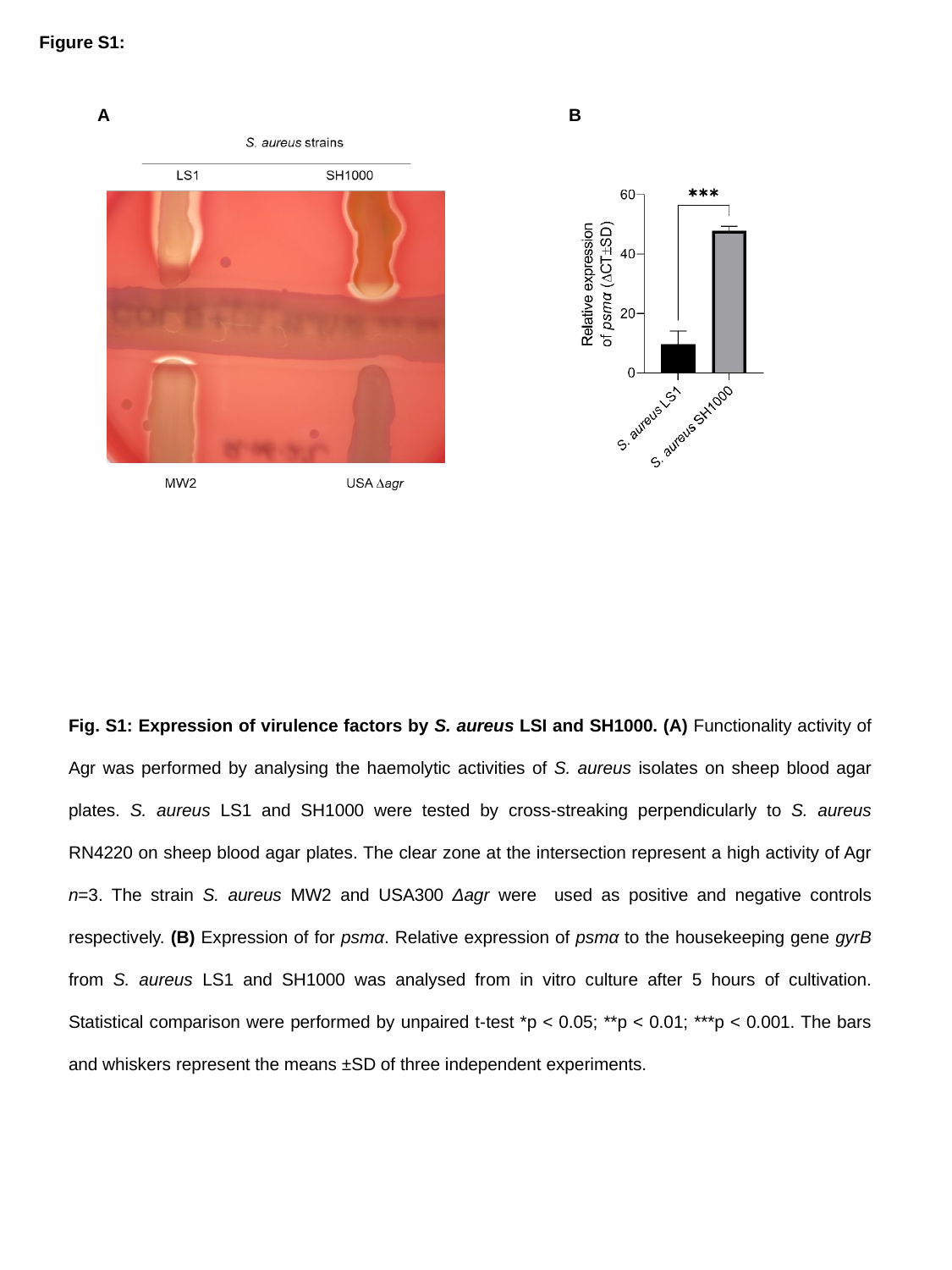

Figure S1:
A
B
Fig. S1: Expression of virulence factors by S. aureus LSI and SH1000. (A) Functionality activity of Agr was performed by analysing the haemolytic activities of S. aureus isolates on sheep blood agar plates. S. aureus LS1 and SH1000 were tested by cross-streaking perpendicularly to S. aureus RN4220 on sheep blood agar plates. The clear zone at the intersection represent a high activity of Agr n=3. The strain S. aureus MW2 and USA300 Δagr were used as positive and negative controls respectively. (B) Expression of for psmα. Relative expression of psmα to the housekeeping gene gyrB from S. aureus LS1 and SH1000 was analysed from in vitro culture after 5 hours of cultivation. Statistical comparison were performed by unpaired t-test *p < 0.05; **p < 0.01; ***p < 0.001. The bars and whiskers represent the means ±SD of three independent experiments.

## Slide 2
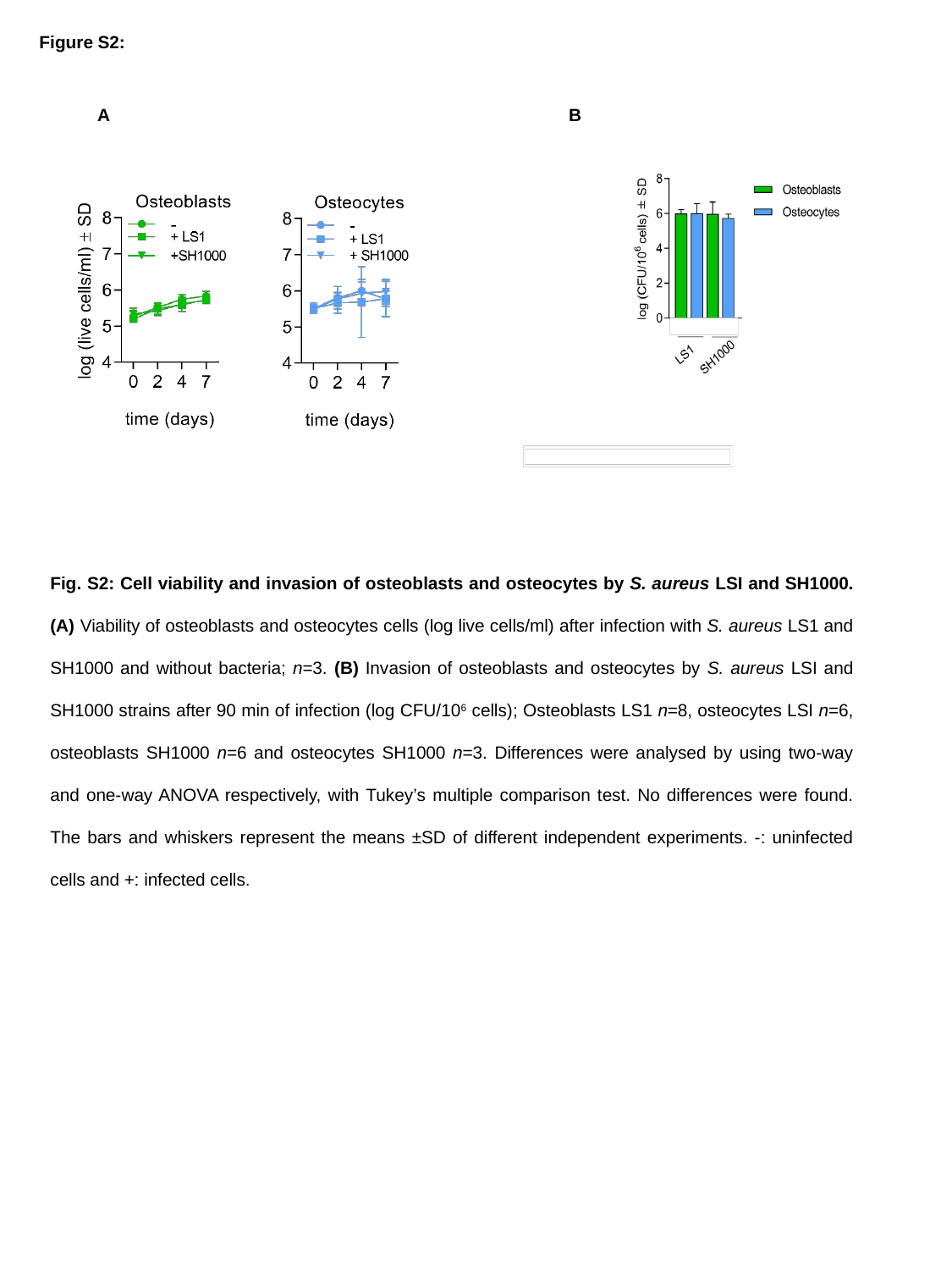

Figure S2:
A
B
Fig. S2: Cell viability and invasion of osteoblasts and osteocytes by S. aureus LSI and SH1000. (A) Viability of osteoblasts and osteocytes cells (log live cells/ml) after infection with S. aureus LS1 and SH1000 and without bacteria; n=3. (B) Invasion of osteoblasts and osteocytes by S. aureus LSI and SH1000 strains after 90 min of infection (log CFU/106 cells); Osteoblasts LS1 n=8, osteocytes LSI n=6, osteoblasts SH1000 n=6 and osteocytes SH1000 n=3. Differences were analysed by using two-way and one-way ANOVA respectively, with Tukey’s multiple comparison test. No differences were found. The bars and whiskers represent the means ±SD of different independent experiments. -: uninfected cells and +: infected cells.

## Slide 3
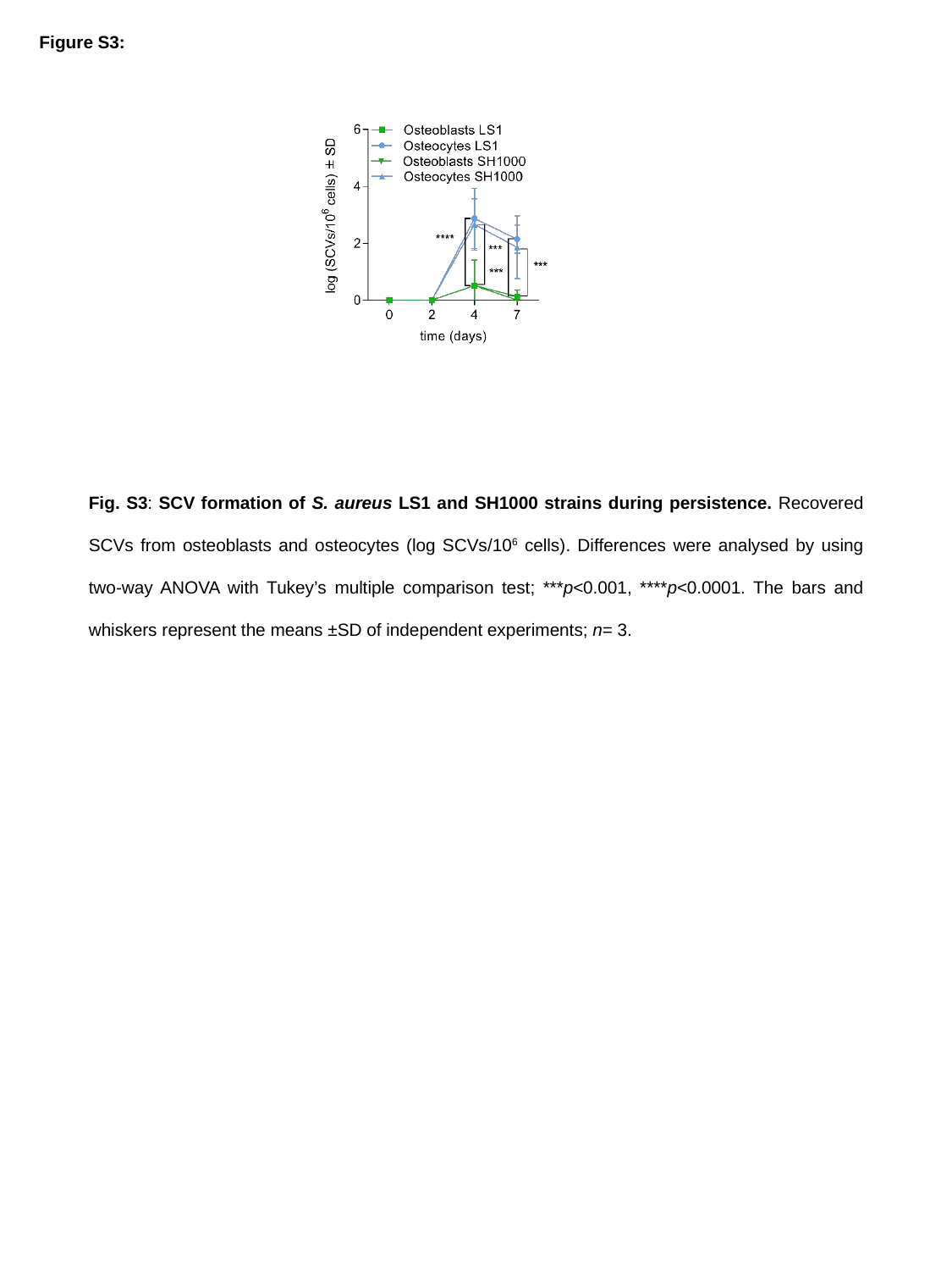

Figure S3:
Fig. S3: SCV formation of S. aureus LS1 and SH1000 strains during persistence. Recovered SCVs from osteoblasts and osteocytes (log SCVs/106 cells). Differences were analysed by using two-way ANOVA with Tukey’s multiple comparison test; ***p<0.001, ****p<0.0001. The bars and whiskers represent the means ±SD of independent experiments; n= 3.

## Slide 4
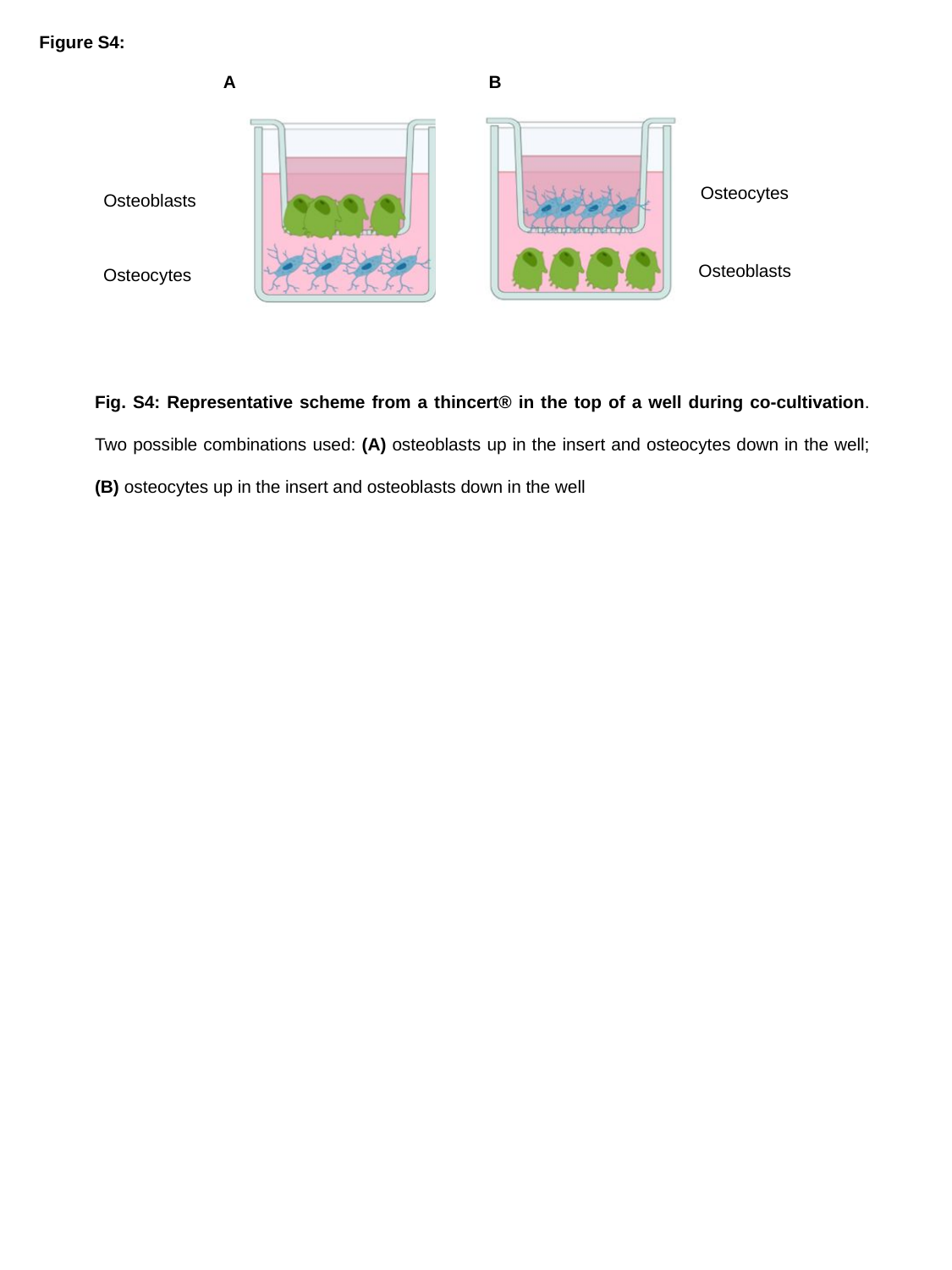

Figure S4:
A
B
Osteocytes
Osteoblasts
Osteoblasts
Osteocytes
Fig. S4: Representative scheme from a thincert® in the top of a well during co-cultivation. Two possible combinations used: (A) osteoblasts up in the insert and osteocytes down in the well; (B) osteocytes up in the insert and osteoblasts down in the well

## Slide 5
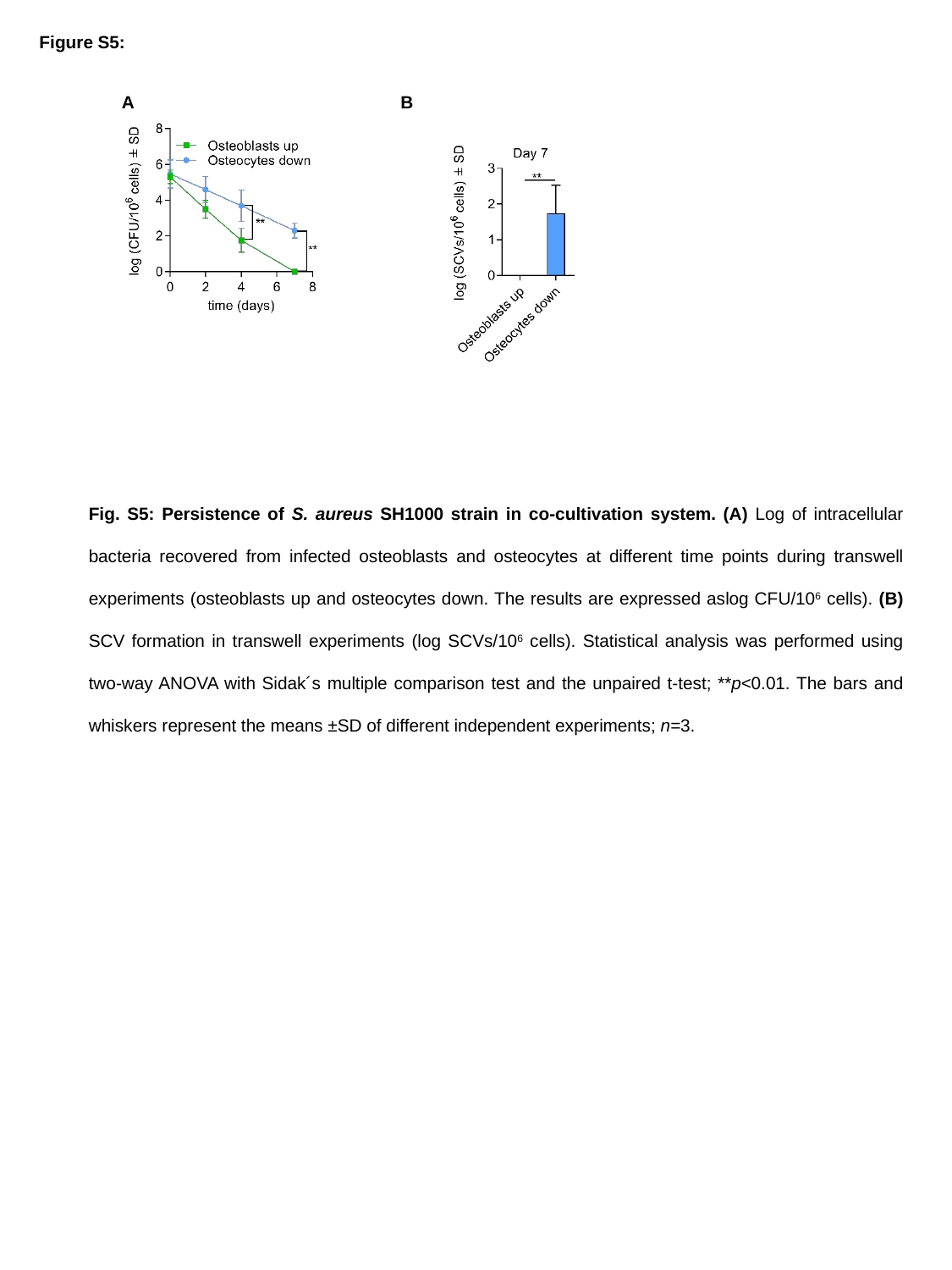

Figure S5:
A
B
Fig. S5: Persistence of S. aureus SH1000 strain in co-cultivation system. (A) Log of intracellular bacteria recovered from infected osteoblasts and osteocytes at different time points during transwell experiments (osteoblasts up and osteocytes down. The results are expressed aslog CFU/106 cells). (B) SCV formation in transwell experiments (log SCVs/106 cells). Statistical analysis was performed using two-way ANOVA with Sidak´s multiple comparison test and the unpaired t-test; **p<0.01. The bars and whiskers represent the means ±SD of different independent experiments; n=3.

## Slide 6
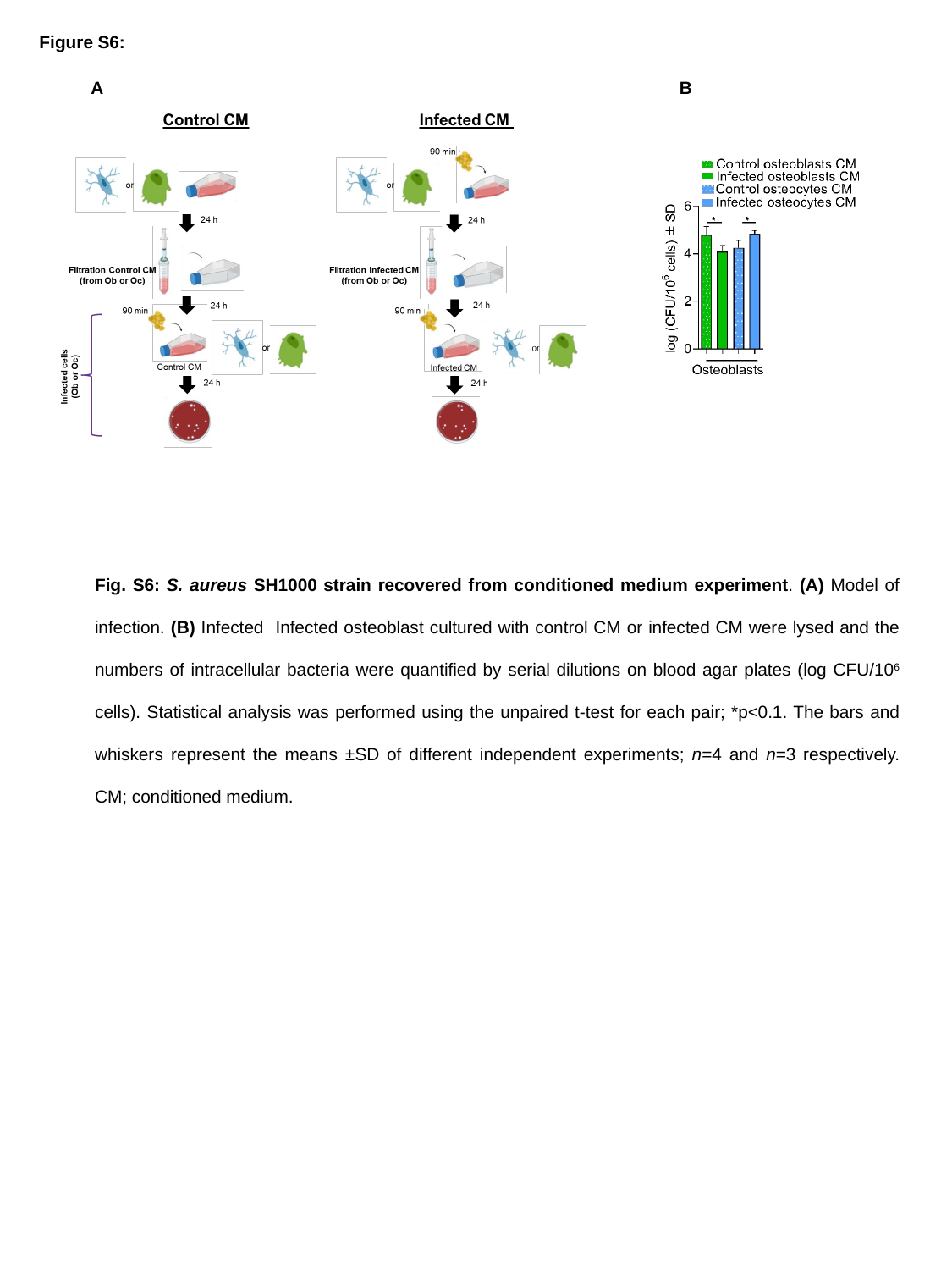

Figure S6:
A
B
Fig. S6: S. aureus SH1000 strain recovered from conditioned medium experiment. (A) Model of infection. (B) Infected Infected osteoblast cultured with control CM or infected CM were lysed and the numbers of intracellular bacteria were quantified by serial dilutions on blood agar plates (log CFU/106 cells). Statistical analysis was performed using the unpaired t-test for each pair; *p<0.1. The bars and whiskers represent the means ±SD of different independent experiments; n=4 and n=3 respectively. CM; conditioned medium.

## Slide 7
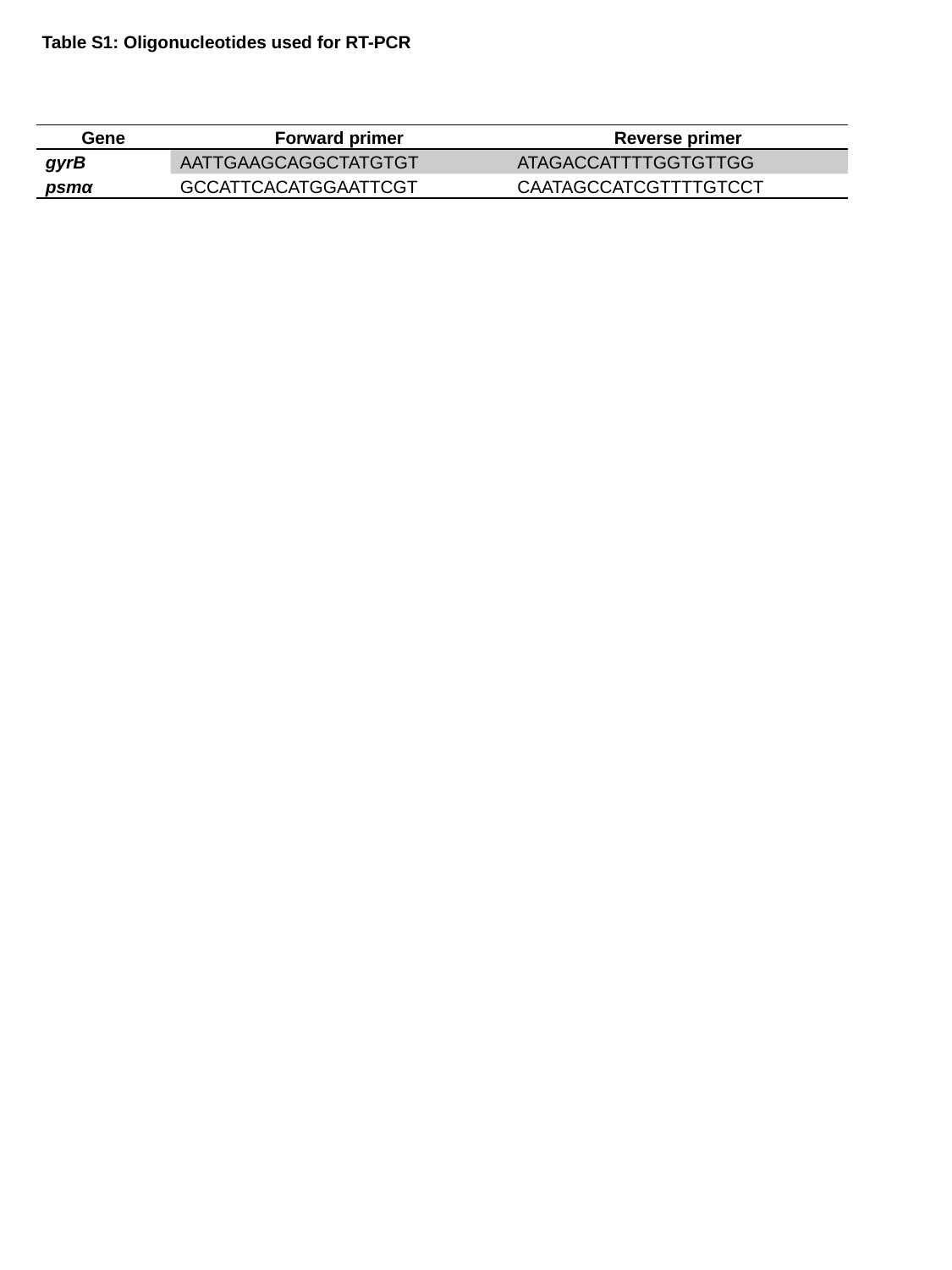

Table S1: Oligonucleotides used for RT-PCR
| Gene | Forward primer | Reverse primer |
| --- | --- | --- |
| gyrB | AATTGAAGCAGGCTATGTGT | ATAGACCATTTTGGTGTTGG |
| psmα | GCCATTCACATGGAATTCGT | CAATAGCCATCGTTTTGTCCT |

## Slide 8
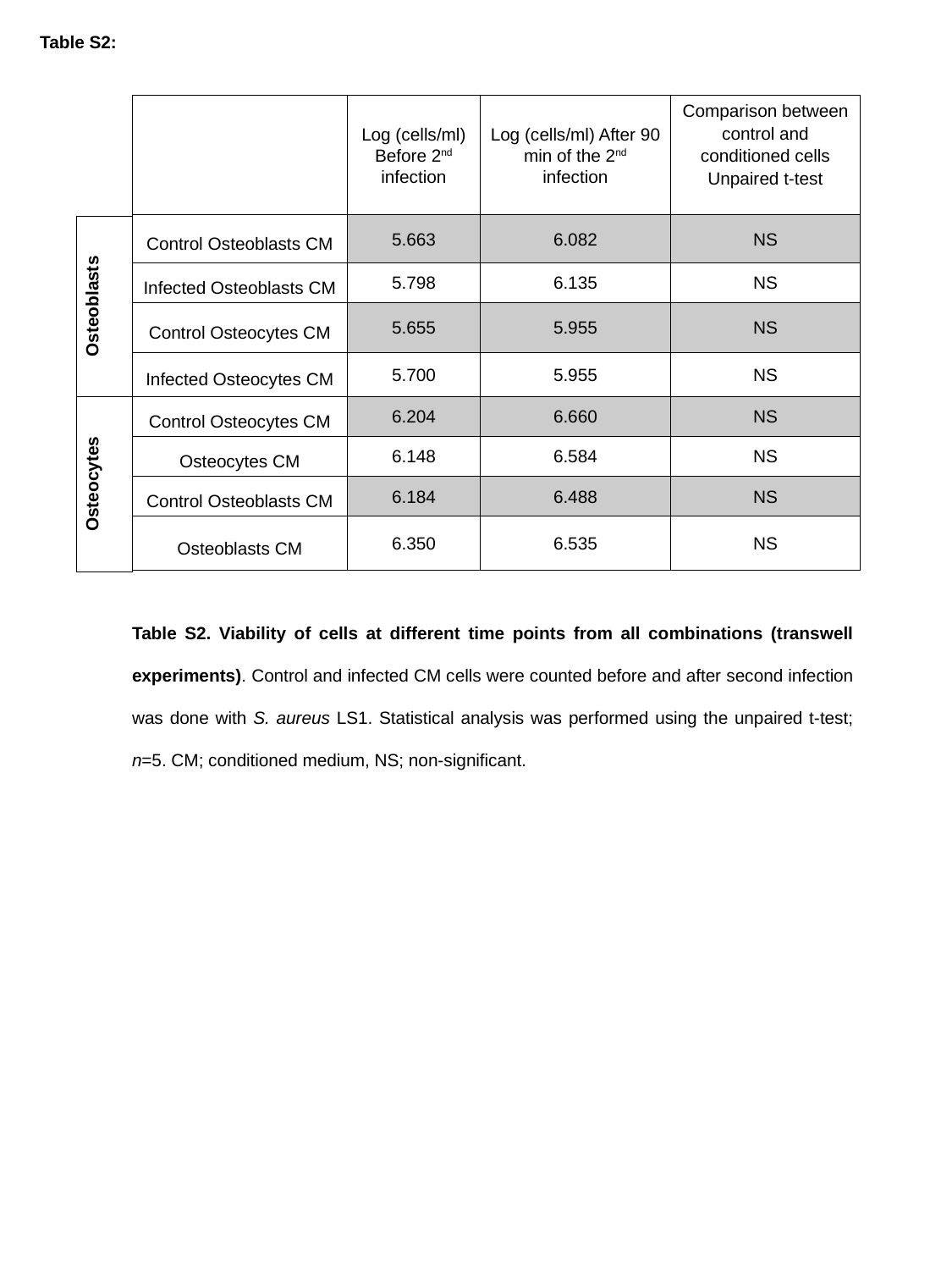

Table S2:
| | Log (cells/ml) Before 2nd infection | Log (cells/ml) After 90 min of the 2nd infection | Comparison between control and conditioned cells Unpaired t-test |
| --- | --- | --- | --- |
| Control Osteoblasts CM | 5.663 | 6.082 | NS |
| Infected Osteoblasts CM | 5.798 | 6.135 | NS |
| Control Osteocytes CM | 5.655 | 5.955 | NS |
| Infected Osteocytes CM | 5.700 | 5.955 | NS |
| Control Osteocytes CM | 6.204 | 6.660 | NS |
| Osteocytes CM | 6.148 | 6.584 | NS |
| Control Osteoblasts CM | 6.184 | 6.488 | NS |
| Osteoblasts CM | 6.350 | 6.535 | NS |
| Osteoblasts |
| --- |
| Osteocytes |
| --- |
Table S2. Viability of cells at different time points from all combinations (transwell experiments). Control and infected CM cells were counted before and after second infection was done with S. aureus LS1. Statistical analysis was performed using the unpaired t-test; n=5. CM; conditioned medium, NS; non-significant.

## Slide 9
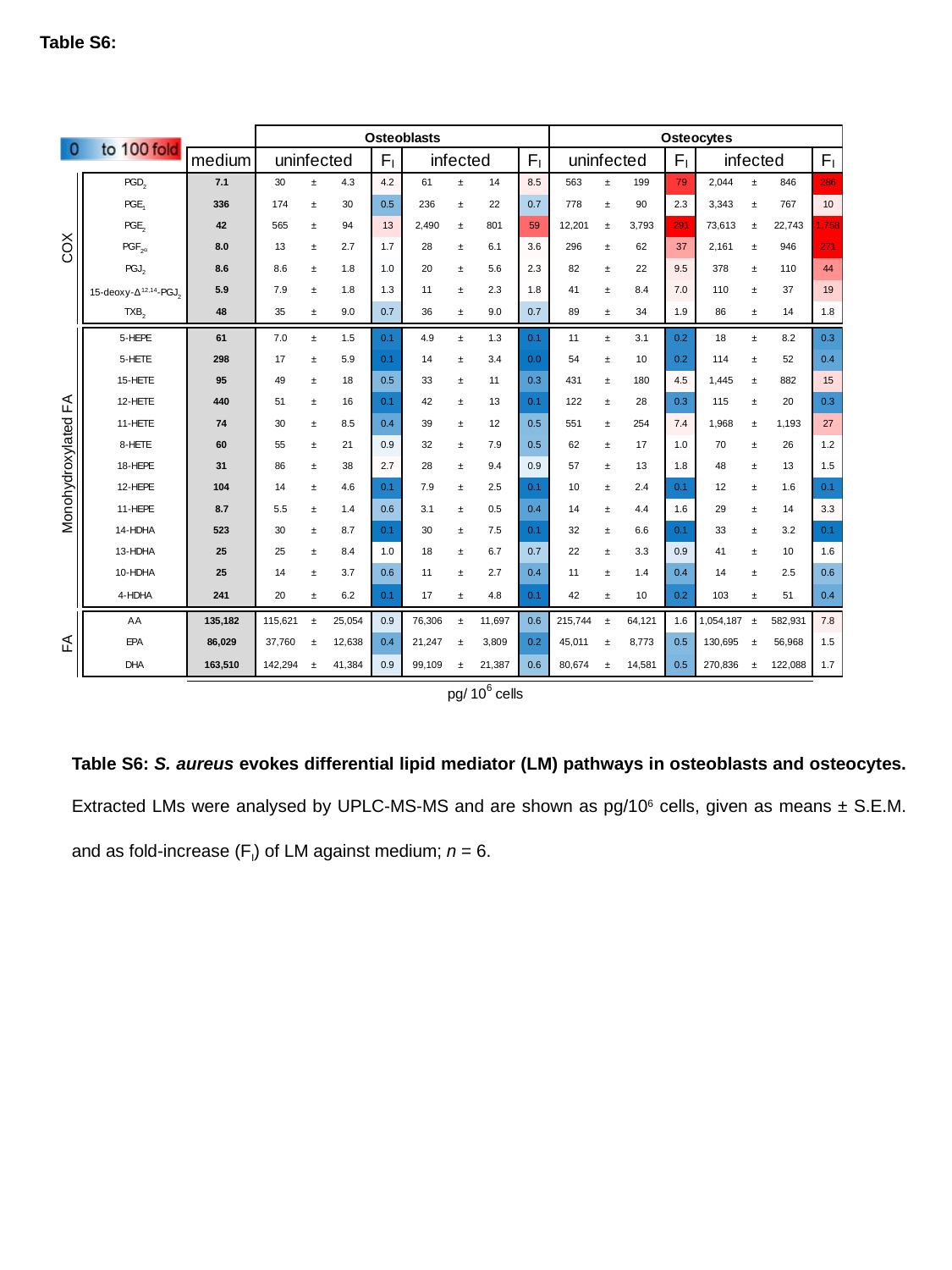

Table S6:
Table S6: S. aureus evokes differential lipid mediator (LM) pathways in osteoblasts and osteocytes. Extracted LMs were analysed by UPLC-MS-MS and are shown as pg/106 cells, given as means ± S.E.M. and as fold-increase (FI) of LM against medium; n = 6.
